# Supplementary material for: Trajectory-based differential expression analysis for single-cell sequencing data
Source: Nat Commun. 2020 Mar 5;11:1201. doi: 10.1038/s41467-020-14766-3 (PMC7058077; doi:10.1038/s41467-020-14766-3)
Supplement: Supplementary file 2 — Supplementary Information [file 41467_2020_14766_MOESM2_ESM.pdf]

Supplementary Information: Trajectory-based differential expression analysis for single-cell sequencing data

Koen Van den Berge[1,2,3], Hector Roux de Bézieux[4], Kelly Street[5], Wouter Saelens[1,6], Robrecht Cannoodt[6,7,8], Yvan Saeys[1,6], Sandrine Dudoit[3,4,*,†], Lieven Clement[1,2,*,†]

1 Department of Applied Mathematics, Computer Science and Statistics, Ghent University, Ghent, Belgium

[2] Bioinformatics Institute Ghent, Ghent University, Ghent, Belgium

[3] Department of Statistics, University of California, Berkeley, CA, USA

[4] Division of Biostatistics, School of Public Health, University of California, Berkeley, CA, USA

[5] Department of Data Sciences, Dana-Farber Cancer Institute, Boston, MA, USA

[6] Data mining and Modelling for Biomedicine, VIB Center for Inflammation Research, Ghent, Belgium

[7] Center for Medical Genetics, Ghent University Hospital, Ghent, Belgium

[8] Department of Biomolecular Medicine, Ghent University, Ghent, Belgium

[*] To whom correspondence should be addressed: sandrine@stat.berkeley.edu, lieven.clement@ugent.be

[†] These authors jointly supervised this work.

January 13, 2020

Supplementary Methods

Eigenvalue decomposition of $\hat{\boldsymbol{\Sigma}}_{\hat{\boldsymbol{\beta}}_g}$

Let $\mathbf{C}$ correspond to the $(LK) \times L(L-1)M/2$ matrix that defines the linear contrasts of interest for the `patternTest`, i.e., every column of $\mathbf{C}$ corresponds to the comparison of two points for a pair of lineages. Tests for the contrasts are performed using a Wald test statistic defined as

$$W_g = \hat{\boldsymbol{\beta}}_g^T \mathbf{C}(\mathbf{C}^T \hat{\boldsymbol{\Sigma}}_{\hat{\boldsymbol{\beta}}_g} \mathbf{C})^{-1} \mathbf{C}^T \hat{\boldsymbol{\beta}}_g,$$

with $\hat{\boldsymbol{\Sigma}}_{\hat{\boldsymbol{\beta}}_g}$ the estimated variance-covariance matrix of the estimated smoother coefficients. Letting $\hat{\boldsymbol{\alpha}}_g = \hat{\boldsymbol{\beta}}_g^T \mathbf{C}$ and $\hat{\boldsymbol{\Sigma}}_{\hat{\boldsymbol{\alpha}}_g} = \mathbf{C}^T \hat{\boldsymbol{\Sigma}}_{\hat{\boldsymbol{\beta}}_g} \mathbf{C}$, we can rewrite the Wald statistic as

$$W_g = \hat{\boldsymbol{\alpha}}_g (\hat{\boldsymbol{\Sigma}}_{\hat{\boldsymbol{\alpha}}_g})^{-1} \hat{\boldsymbol{\alpha}}_g^T.$$

Taking the eigendecomposition of $\hat{\boldsymbol{\Sigma}}_{\hat{\boldsymbol{\alpha}}_g}$,

$$W_g = \hat{\boldsymbol{\alpha}}_g \hat{\mathbf{V}}_g^T \hat{\boldsymbol{\Lambda}}_g^{-1} \hat{\mathbf{V}}_g \hat{\boldsymbol{\alpha}}_g^T,$$

where $\hat{\mathbf{V}}_g$ is an $L(L-1)M/2 \times L(L-1)M/2$ matrix with columns corresponding to the $L(L-1)M/2$ eigenvectors of $\hat{\boldsymbol{\Sigma}}_{\hat{\boldsymbol{\alpha}}_g}$ and $\hat{\boldsymbol{\Lambda}}_g$ the $L(L-1)M/2 \times L(L-1)M/2$ diagonal matrix of eigenvalues $\lambda_i \in [0,1]$ of $\hat{\boldsymbol{\Sigma}}_{\hat{\boldsymbol{\alpha}}_g}$, in decreasing order. Note that, since $\hat{\boldsymbol{\Lambda}}_g$ is a diagonal matrix, we can simply invert its diagonal elements instead of inverting the full matrix. We determine the rank r of $\hat{\boldsymbol{\Sigma}}_{\hat{\boldsymbol{\alpha}}_g}$ by calculating the number of eigenvalues that are larger than $1e^{-8}\lambda_1$, with λ_1 corresponding to the largest eigenvalue. When the rank r of $\hat{\boldsymbol{\Sigma}}_{\hat{\boldsymbol{\alpha}}_g}$ is less than $L(L-1)M/2$ (i.e., the matrix is not of full rank), we only use the first r eigenvectors from $\hat{\mathbf{V}}_g$, associated with the largest r eigenvalues from $\hat{\boldsymbol{\Lambda}}_g$. This provides an efficient computation of the test statistic and avoids singularity problems with the estimated variance-covariance matrix of the contrasts[1].

Defining $\mathbf{Z}$ based on user-supplied weights

If one has user-supplied weights $\mathbf{W} = (W_{li} \in [0,1] : l \in \{1,\ldots,L\}, i \in \{1,\ldots,n\})$ for the assignment of cells to lineages, one can construct the binary matrix $\mathbf{Z}$ from $\mathbf{W}$ as follows.

First, note that the weights $\mathbf{W}$ may be defined differently depending on the TI method that was used to estimate them. For example, slingshot[2] defines weights based on the distance from a cell to a particular lineage; hence, the sum of the weights across all lineages for a particular cell may be greater than 1. As such, these weights cannot be interpreted as probabilities. GPfates[3], however, does return posterior probabilities that a cell i belongs to a particular lineage l, where $\sum_{l=1}^{L} W_{li} = 1$ for each i. We therefore first normalize the weights for each cell, such that, for normalized weights W^*_{li}, the sum across lineages equals one, i.e., $\sum_{l=1}^{L} W^*_{li} = 1$ for each cell i. Next, we assign each cell i to a lineage by sampling one observation from a multinomial distribution with L groups and probabilities W^*_{li}. The lineage assignments are then encoded in the $L \times n$ matrix $\mathbf{Z}$, by setting all elements of the ith column equal to zero except for a 1 in the row corresponding to the sampled lineage for cell i.

The multinomial sampling to assign each cell to a lineage may introduce variability in the results if the models are fit multiple times, due to differing cell allocations. This is especially so if there is a high uncertainty about the lineage allocation (e.g., a cell is equally likely to belong to each of two lineages), which typically occurs around the inception of a trajectory. While we ensure computational reproducibility by setting a seed in the software implementation, the results may vary slightly over different seeds. To quantify this variability, we use the data of Paul et al.[4] and allocate cells to lineages using 10 different seeds. Since we expect the variability across different assignments to be largest at the inception of the lineage, we evaluate DE using tradeSeq's `startVsEndTest`. Using a global test across the two lineages, the number of DE genes at a 5% nominal FDR level varied between 1,990 and 2,049, with 1,739 DE genes shared across all 10 assignments (Supplementary Figure 31). For each assignment, at least 93% of the top 1,000 DE genes are shared with the top 1,000 DE genes of any other assignment.

Supplementary Note 1: Computation time and memory usage benchmark

To assess time and memory requirements, scRNA-seq datasets with a bifurcating trajectory were simulated using the same framework as in the simulation study. Three datasets with 100, $1,000$, and $10,000$ cells were simulated (small, medium, and large datasets), each consisting of $5,000$ genes. For BEAM and GPfates, only the fitting and DE testing part was assessed for each method, not the trajectory inference part. All methods were ran with default options. For tradeSeq, the `fitGAM` function was assessed with 4 knots, as determined in the simulation study. The different tests implemented in tradeSeq were benchmarked separately (Supplementary Figure 16). Their running times are very small (always below 30 seconds), as compared to the `fitGAM` function, and do not increase for datasets with increasing numbers of cells.

To benchmark time requirements, the microbenchmark package was used, and each method was run 10, 2, and 2 times on, respectively, the small, medium, and large datasets. Variations in running times were very small (always under the minute), especially in comparison to between method differences. Methods that reached the 4-hour mark without finishing were killed. This is the case for ImpulseDE2 on the datasets with 10^3 and 10^4 cells, and for GPfates on the largest dataset. Memory benchmark was assesses using the `Rprof` function; maximum memory usage was recorded.

Results are shown in Figure 4. ImpulseDE2 is by far the slowest, taking over 3.5 hours to finish on a small dataset of 100 cells. GPfates runs in about 30s on the small dataset but scales poorly. BEAM, edgeR, and tradeSeq are quite fast and scale very well, even to large datasets, with BEAM scaling the best. In terms of memory requirements, all methods scale well to $10,000$ cells. It should also be noted that tradeSeq, ImpulseDE2, and BEAM can utilize multiple cores but were benchmarked using only one core.

Supplementary Note 2: Bulk RNA-seq time-course dataset

While in this manuscript we focus on DE analysis downstream of TI, the applicability of `tradeSeq` extends beyond this setting. We demonstrate this by using `tradeSeq` on a bulk RNA-seq time-course study from Kiselev et al.[5], where we compare gene expression between wild type and PIK3CA H1047R cell lines upon stimulation of epidermal growth factor (EGF). Gene expression was measured for three replicates in each condition over six time-points, ranging from 0 to 300 minutes post EGF stimulation. The original analysis in the manuscript assessed DE between the cell lines for each time-point separately using `DESeq2`, and found $7,486$ DE genes at a 1% nominal FDR level (Benjamini-Hochberg[6] FDR-controlling procedure). We perform an analogous analysis using `tradeSeq`, by modeling gene expression measures as smooth functions of time and looking for differences in expression patterns with `patternTest`. This yields $7,184$ DE genes at a 1% nominal FDR level. Around 89% of these genes overlap with the original DE list of $7,486$ genes, demonstrating that the utility of `tradeSeq` goes beyond scRNA-seq applications.

Supplementary Note 3: Adipocyte differentiation dataset

As final case study, we reanalyze a 10x Genomics scRNA-seq dataset from Merrick et al.[7], studying adipocyte differentiation from the developing sub-cutaneous inguinal white adipose tissue (iWAT) of 12-day-old mice. We use tradeSeq to fit NB-GAMs with 8 knots (Supplementary Figure 17) based on the trajectory inferred by slingshot in 2-dimensional (2D) UMAP space. As in the original manuscript, the progenitor cells differentiate into two different cell populations (Supplementary Figure 18). While we confirm *Dpp4+* and *Wnt2* as interstitial progenitor markers, we discover several other markers as top genes from our `startVsEndTest` procedure that are even more pronounced, e.g., *Pi16*, *Akr1c18*, *Fn1*, and *Fbn1* (Supplementary Figure 19). In addition, we search for markers distinguishing between the two differentiated cell populations. Since these are relatively large heterogeneous groups of cells, `diffEndTest` is not representative for the entire set of cells. However, `earlyDETest` can be used to discover DE across their developmental range. This reveals several interesting patterns, such as genes upregulated in the adipocyte precursor stage and subsequently downregulated in only a single differentiated cell population (e.g., *Mgp* and *Meox2*; Supplementary Figure 20), as well as genes that are sporadically highly expressed across the entire lineage for one of the two differentiated cell populations (e.g., *H19* and *Col14a1*; Supplementary Figure 21)).

Supplementary Note 4: Mouse bone marrow dataset

Dimensionality reduction. We observed that the biology of the ICA dimensionality reduction does not fully preserve the underlying biology. Indeed, a 2-dimensional visualization of the ICA dimensionality reduction (Figure 5a) shows that there is a seemingly large gap between the multipotent progenitors and the remaining cell types, and that a number of erythrocytes and granulocyte-macrophage progenitors (GMP) are misclassified as multipotent progenitors. In addition, megakaryocytes, which are thrombocyte progenitors and as such should not belong to any of the two lineages, seem to be split between the erythrocyte and leukocyte lineages. However, when applying UMAP dimensionality reduction (Figure 5b), these issues are resolved and the underlying biology seems better preserved than with ICA.

Discovering cell type markers. The `diffEndTest` procedure from tradeSeq finds 2, 233 significantly differentially expressed genes at a 5% nominal FDR level,while BEAM discovers 584 genes at a 5% nominal FDR level when testing whether the association between gene expression and pseudotime depends on the lineage (Benjamini-Hochberg [6] FDR-controlling procedure). Since the identification of a larger set of DE genes does not necessarily imply more relevant biology, we select carefully constructed gene sets from de Graaf et al. [8] to perform gene set enrichment analysis (GSEA) on blood cell types. As we are comparing erythrocytes with a mixture of leukocytes, we expect gene sets related to erythrocytes to be significant. Indeed, the erythrocyte gene set is the only one to be found significant by fgsea [9] for the tradeSeq analysis (FDR adjusted p-value $< .001$, with normalized enrichment score of 1.49), while no significant gene sets are found for the BEAM analysis (as reference, the FDR adjusted p-value for the erythrocyte gene set is 0.58). In this case, tradeSeq is therefore better able to recover a meaningful biological signal (Supplementary Figure 24). If one assumes that the cell type labels are known for all cells in the dataset, a cluster-based comparison is possible, where the different clusters correspond to the identified cell types. We use edgeR [10] to assess differential expression between erythrocytes and neutrophils, since this comparison is most analogous to tradeSeq's `diffEndTest`. Only edgeR finds evidence for gene sets related to eosinophils and T-cells (FDR adjusted p-values of 0.042 and 0.049, respectively), however, the eosinophil cells were removed from this dataset prior to analysis (see Methods, subsection 'Case studies: Mouse bone marrow dataset'). The GSEA results for edgeR also provide less evidence for erythrocytes (FDR adjusted p-value of 0.043, normalized enrichment score of 1.21) as compared to the tradeSeq analysis (Supplementary Figure 24). None of the methods, however, recover evidence for the neutrophil cell types that are identified at the end of the lineage (Figure 5b; FDR adjusted p-values $p_{\text{tradeSeq}} = 0.99$, $p_{\text{BEAM}} = 0.86$, and $p_{\text{edgeR}} = 0.81$).

Discovering progenitor population markers. It might be interesting to examine genes with significantly different expression patterns, that show little evidence for DE at the endpoints. We therefore select genes with both a high Wald test statistic (low p-value) for the `patternTest` and a low test statistic (high p-value) for the `diffEndTest`. Following the approach described in 'Case studies: Mouse bone marrow dataset' (Methods), we assign a score for each gene. Remarkably, within the top eight genes, four genes (*Erp29*, *Irf8*, *Psap*, and *ApoE*) were previously found to be major regulators of hematopoiesis. Indeed, *Irf8* has previously been identified as a major transcription factor involved in myeloid lineage commitment [4,11]. *Erp29* and *Psap* are direct targets of the *Irf8* transcription factor [4,12,13], while *ApoE* regulates stem cell proliferation in atherosclerotic mice [14] and was also identified as a marker gene in the original manuscript of Paul et al. [4]. The remaining four genes include *Nedd4*, *Srrm2*, *Gatm*, and *Acin1*.

Supplementary Note 5: Mouse olfactory epithelium dataset

Within-lineage DE. The top 20 gene sets as derived by the `startVsEndTest` procedure across all three lineages in a ZINB-tradeSeq analysis confirms the biology of the experiment. The HBCs were primed for differentiation and the top gene sets include responses to (organic, external, and endogenous) stimuli. In addition, neurogenesis and tissue development are the first and third most significant gene sets, respectively, while the remaining list contains sets related to cell development, differentiation, and epithelium development, amongst others. Although the neuronal and microvillous cell lineages undergo mitotic division, it is worth noting that cell cycle related gene sets are absent from the `startVsEndTest` results, since this process occurs during differentiation, but not in the resting HBC or differentiated cell populations.

Between-lineage DE. The only relevant comparison is between the `diffEndTest` procedure from tradeSeq and a discrete DE test between the differentiated cell types using ZINB-edgeR as introduced in Van den Berge et al.[15]. For both methods, we use a global test to compare mean expression between all three differentiated cell types. While a ZINB-edgeR analysis discovers $1,984$ genes, the ZINB-tradeSeq analysis discovers $3,719$ genes, which include $\sim 86\%$ of the genes also discovered by the ZINB-edgeR analysis. In order to assess the relevance of the extra $1,994$ genes discovered with ZINB-tradeSeq, we perform GSEA on this gene set (Supplementary Table 3). The top 20 significant gene sets contain relevant biological processes for the system under study, such as "regulation of multicellular organismal development", "positive regulation of biosynthetic process", and "tissue development".

We can also identify genes that drive the branching based on the `earlyDETest` applied around the first branching point, i.e., between knots 1 and 3 (see Supplementary Figure 29 and Figure 6d). We apply stage-wise testing[16] (see Methods) to first assess any difference across the three lineages using a global test. We discover $2,083$ genes to be DE between any of the three lineages at a 5% nominal FDR level. Among these $2,083$ genes, we then discover 634 significant genes between the neuronal and microvillous lineages, $1,068$ significant genes between the microvillous and sustentacular lineages, and $1,312$ significant genes between the neuronal and sustentacular lineages (Supplementary Figure 30). In total, 151 genes are significant in all pairwise comparisons (Supplementary Figure 30), and these genes may be potentially important regulators of the transcriptional program involved in olfactory epithelium development. In these early stages of development, one could expect transcription factors to drive the differences between the three developmental lineages. Out of all $2,083$ significant genes, we recover 84 transcription factors, as identified by TFcheckpoint[17]. Interestingly, aside from their general functionality as regulators of gene expression, the list of 84 transcription factors is enriched for gene sets related to epithelial cell differentiation, cell fate commitment, neuron differentiation, amongst other relevant gene sets (Supplementary Table 4).

Supplementary Tables and Figures

	Gene set	Overlap	Genes in set	q-value
1	neurogenesis	46	1402	1.31E-21
2	response to external stimulus	51	1821	1.41E-21
3	tissue development	47	1518	1.41E-21
4	cellular response to organic substance	49	1848	7.41E-20
5	regulation of multicellular organismal development	46	1672	3.5E-19
6	neuron differentiation	35	874	3.69E-19
7	response to endogenous stimulus	43	1450	4.07E-19
8	regulation of cell differentiation	43	1492	1.05E-18
9	regulation of cellular component movement	32	771	5.84E-18
10	cell development	41	1426	8.94E-18
11	cellular response to endogenous stimulus	35	1008	1.91E-17
12	regulation of intracellular signal transduction	43	1656	3.5E-17
13	organ morphogenesis	31	841	4.86E-16
14	negative regulation of response to stimulus	38	1360	4.86E-16
15	positive regulation of cell communication	40	1532	5.3E-16
16	regulation of phosphorus metabolic process	40	1618	3.24E-15
17	response to oxygen containing compound	37	1381	4.61E-15
18	circulatory system development	29	788	5.19E-15
19	epithelium development	31	945	8.78E-15
20	locomotion	33	1114	1.44E-14

Supplementary Table 1: *Mouse olfactory epithelium dataset.* The top 20 significant GO sets for the top 250 genes when assessing global differential expression between the progenitor and differentiated cell populations using the `tradeSeq` `startVsEndTest` procedure. The "Overlap" column records the number of genes, out of the 250 top genes, that are included in a particular gene set. The significance of a gene set is measured by a q-value, assessing the significance of the overlap of the DE genes with the gene set, as obtained from the Molecular Signatures Database v6.2 (http://software.broadinstitute.org/gsea/msigdb).

	Gene set	Overlap	Genes in set	q-value
1	cell cycle	121	1316	3.97E-57
2	cell cycle process	108	1081	2.66E-54
3	mitotic cell cycle	86	766	1.16E-46
4	establishment of localization in cell	108	1676	2.37E-36
5	cell division	59	460	1.33E-34
6	chromosome organization	79	1009	1.3E-31
7	cellular response to stress	97	1565	3.59E-31
8	organonitrogen compound metabolic process	104	1796	3.59E-31
9	regulation of cell cycle	75	949	2.53E-30
10	organelle fission	54	496	3.91E-28
11	cytoskeleton organization	68	838	4.64E-28
12	microtubule based process	55	522	4.9E-28
13	cellular catabolic process	84	1322	1.31E-27
14	regulation of cell differentiation	89	1492	2.03E-27
15	neurogenesis	86	1402	2.68E-27
16	catabolic process	97	1773	3.09E-27
17	mitotic nuclear division	46	361	6.11E-27
18	positive regulation of molecular function	96	1791	2.65E-26
19	protein complex subunit organization	88	1527	3.81E-26
20	positive regulation of gene expression	94	1733	3.94E-26

Supplementary Table 2: *Mouse olfactory epithelium dataset.* The top 20 significant GO sets for the 827 genes that were found to be significant in all pairwise comparisons between the three trajectories using the `tradeSeq patternTest` procedure. The "Overlap" column records the number of genes, out of the 827 top genes, that are included in a particular gene set. The significance of a gene set is measured by a q-value, assessing the significance of the overlap of the DE genes with the gene set, as obtained from the Molecular Signatures Database v6.2 (http://software.broadinstitute.org/gsea/msigdb).

	Gene set	Overlap	Genes in set	q-value
1	phosphate containing compound metabolic process	225	1977	3.42E-53
2	protein localization	211	1805	8.95E-52
3	regulation of anatomical structure morphogenesis	154	1021	5.52E-51
4	positive regulation of molecular function	207	1791	3.41E-50
5	positive regulation of catalytic activity	186	1518	1.59E-48
6	regulation of multicellular organismal development	193	1672	1.34E-46
7	single organism biosynthetic process	169	1340	1.29E-45
8	lipid metabolic process	153	1158	1.35E-43
9	small molecule metabolic process	193	1767	3.5E-43
10	positive regulation of biosynthetic process	194	1805	1.95E-42
11	positive regulation of gene expression	189	1733	3.31E-42
12	regulation of protein modification process	187	1710	6.25E-42
13	positive regulation of response to stimulus	199	1929	4.54E-41
14	cellular macromolecule localization	154	1234	5.03E-41
15	regulation of phosphorus metabolic process	178	1618	3.29E-40
16	catabolic process	187	1773	7.94E-40
17	intracellular signal transduction	174	1572	1.2E-39
18	regulation of response to stress	167	1468	1.59E-39
19	regulation of transcription from rna polymerase ii promoter	187	1784	1.59E-39
20	tissue development	170	1518	2.08E-39

Supplementary Table 3: *Mouse olfactory epithelium dataset.* The top 20 significant GO sets based on the unique $1,994$ genes that were only discovered with the ZINB-`tradeSeq` analysis with the `diffEndTest` procedure, and not the ZINB-`edgeR` analysis, when comparing mean expression between the endpoints of the lineages. The "Overlap" column records the number of genes, out of the $1,994$ top genes, that are included in a particular gene set. The significance of a gene set is measured by a q-value, assessing the significance of the overlap of the DE genes with the gene set, as obtained from the Molecular Signatures Database v6.2 (http://software.broadinstitute.org/gsea/msigdb).

	Gene set	Overlap	Genes in set	q-value
1	regulation of transcription from RNA Pol. II promoter	68	1784	1.01E-77
2	positive regulation of gene expression	57	1733	6.09E-58
3	positive regulation of biosynthetic process	57	1805	4.12E-57
4	positive regulation of transcription from RNA Pol. II promoter	48	1004	1.06E-54
5	transcription from RNA Pol. II promoter	40	724	1.01E-46
6	negative regulation of transcription from RNA Pol. II promoter	34	740	3.94E-36
7	negative regulation of gene expression	40	1493	2.33E-34
8	negative regulation of nitrogen compound metabolic process	40	1517	3.8E-34
9	tissue development	33	1518	1.42E-24
10	epithelium development	25	945	7.63E-20
11	muscle structure development	19	432	1.24E-18
12	epithelial cell differentiation	19	495	1.46E-17
13	regulation of cell differentiation	27	1492	1.55E-17
14	cell fate commitment	15	227	4.56E-17
15	embryo development	22	894	1.18E-16
16	cell development	25	1426	8.58E-16
17	neuron differentiation	20	874	2.14E-14
18	neurogenesis	23	1402	9.12E-14
19	positive regulation of cell differentiation	19	823	1.07E-13
20	positive regulation of developmental process	21	1142	2.11E-13

Supplementary Table 4: *Mouse olfactory epithelium dataset.* The top 20 significant GO sets based on the 84 transcription factors that were discovered with `earlyDETest` around the branching of the OE trajectory. The "Overlap" column records the number of genes, out of the 1,994 top genes, that are included in a particular gene set. The significance of a gene set is measured by a q-value, assessing the significance of the overlap of the DE genes with the gene set, as obtained from the Molecular Signatures Database v6.2 (http://software.broadinstitute.org/gsea/msigdb).

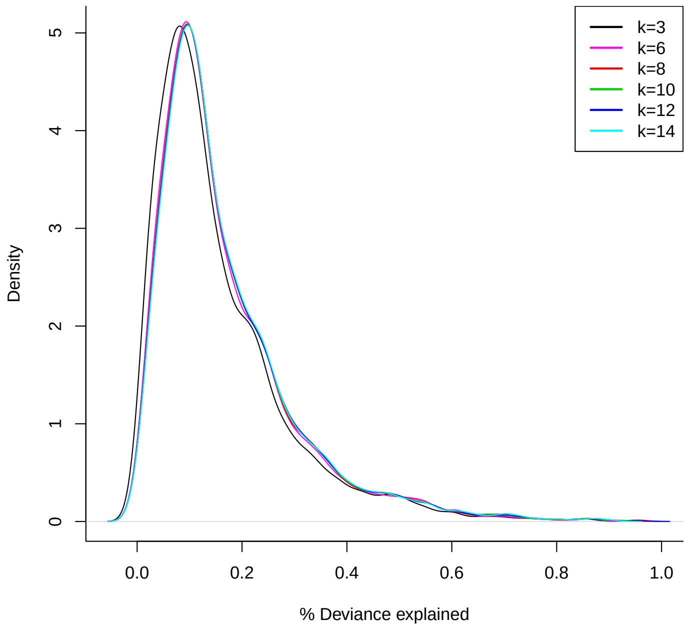

Supplementary Figure 1: *Mouse bone marrow dataset: The NB-GAM is robust to the number of knots k.* Gaussian kernel density plot of the percentage of deviance explained by the NB-GAM applied to each of the genes in the dataset from Paul et al. [4], with number of knots k ranging from 3 to 14. The distributions are nearly identical for the different numbers of knots, except for 3 knots, suggesting we might want to select more than 3 knots for this dataset.

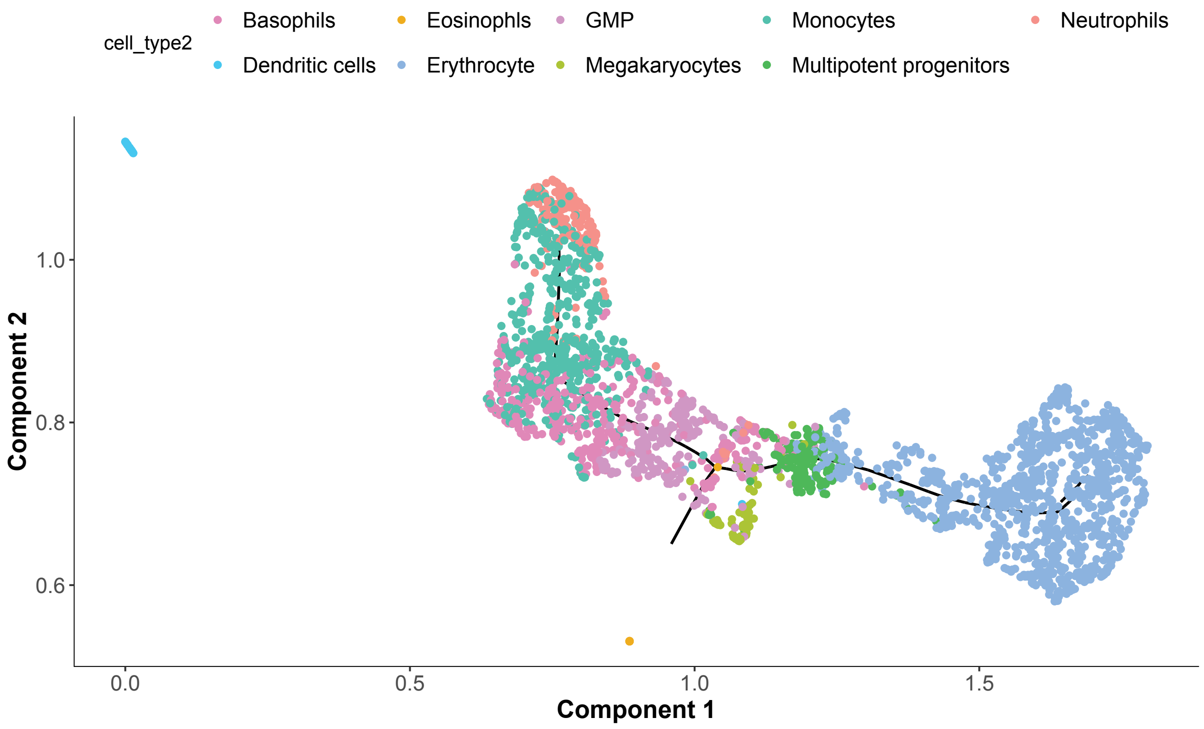

Supplementary Figure 2: *Mouse bone marrow dataset: Outlying dendritic cells and eosinophils in UMAP space for TI with Monocle 3.*

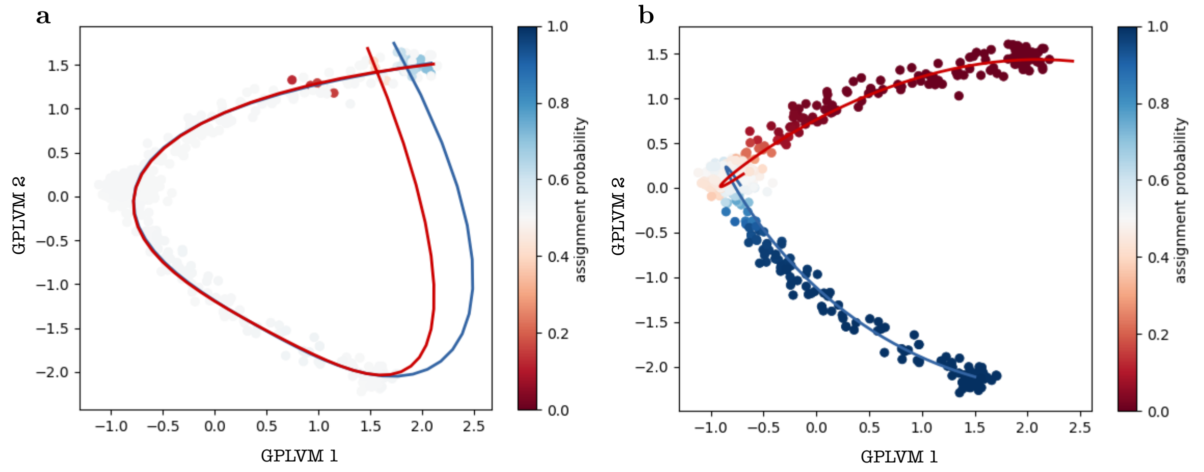

Supplementary Figure 3: *Bifurcating simulation scenario: GPfates only recovers meaningful trajectories if the true pseudotime is provided as input.* Example of a bifurcating dataset from the dynverse framework. The dataset is represented in low-dimensional space using Gaussian process latent variable models as implemented in GPfates. Cells are colored according to their assignment probability to the blue lineage. Trajectories inferred by GPfates are shown when **(a)** pseudotime is estimated by GPfates and **(b)** true pseudotime is provided as input to GPfates.

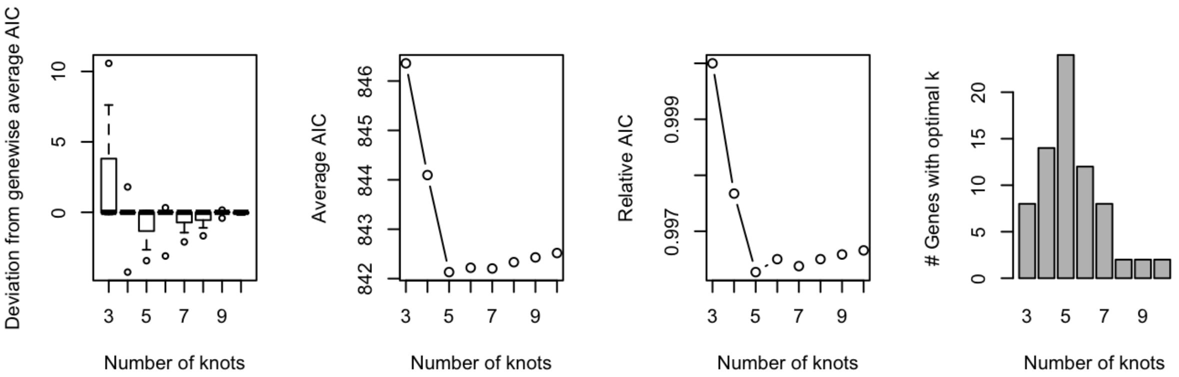

Supplementary Figure 4: *Cyclic simulation scenario: Selecting the optimal number of knots k using the AIC.* Selecting the optimal number of knots, $k \in \{3, \ldots, 10\}$, using the Akaike information criterion (AIC) for a random subset of 250 genes, as implemented in the `evaluateK` function in `tradeSeq`. The left panel shows boxplots (center line, median; box limits, upper and lower quartiles; whiskers, 1.5× interquartile range) of the differences in AIC value with respect to the gene-wise average AIC for the range of k. The middle panels show the evolution of the average AIC (second panel) and relative AIC (third panel) across k. The relative AIC is defined as the relative change with respect to the average AIC at $k = 3$. The barplot in the right panel shows the number of genes which achieve their lowest AIC value for a given k. Here, only genes for which the AIC value varied substantially enough across k (i.e., range in AIC greater than 2) are considered.

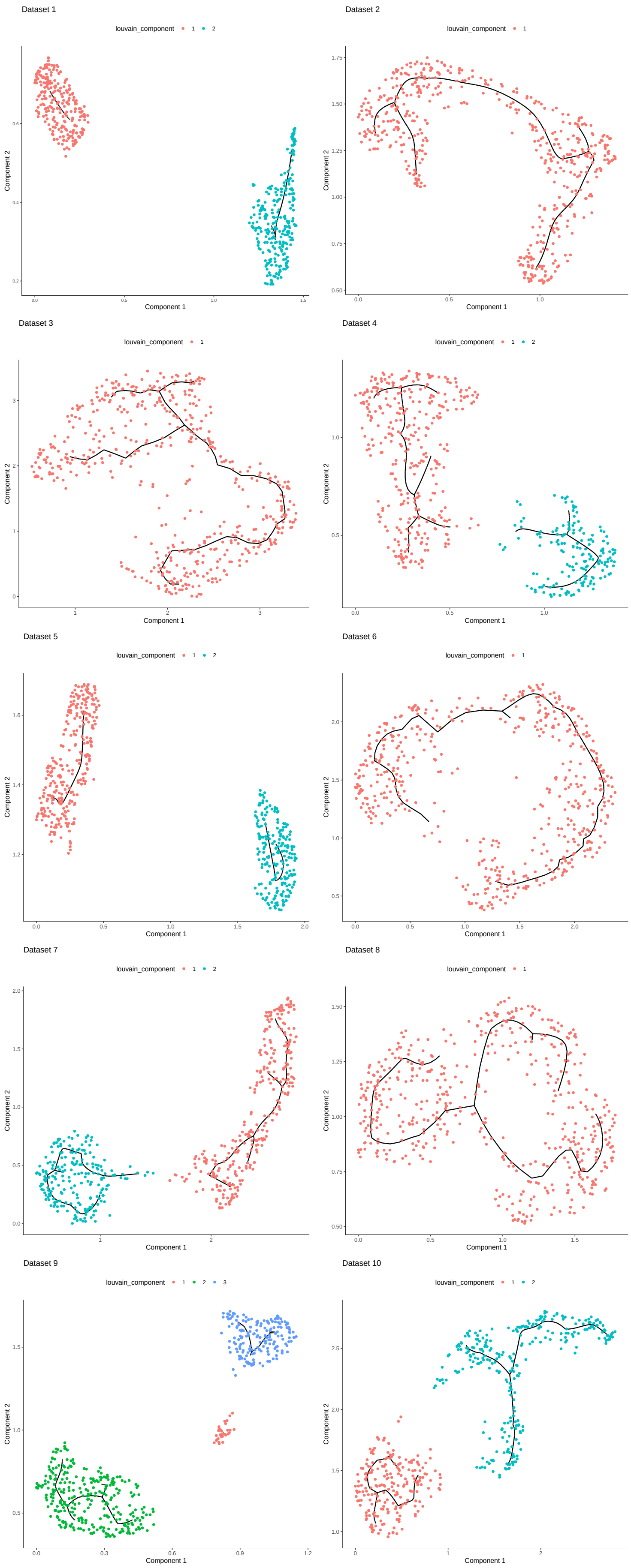

Supplementary Figure 5: *Cyclic simulation scenario: Monocle 3 inferred trajectories for each of the* 10 *simulated datasets.* The first two components from UMAP dimensionality reduction, as implemented in Monocle 3, are plotted along with the Monocle 3 inferred trajectories. Cells are colored according to a Louvain clustering implemented in Monocle 3. Monocle 3 often fails to recover the cyclic pattern.

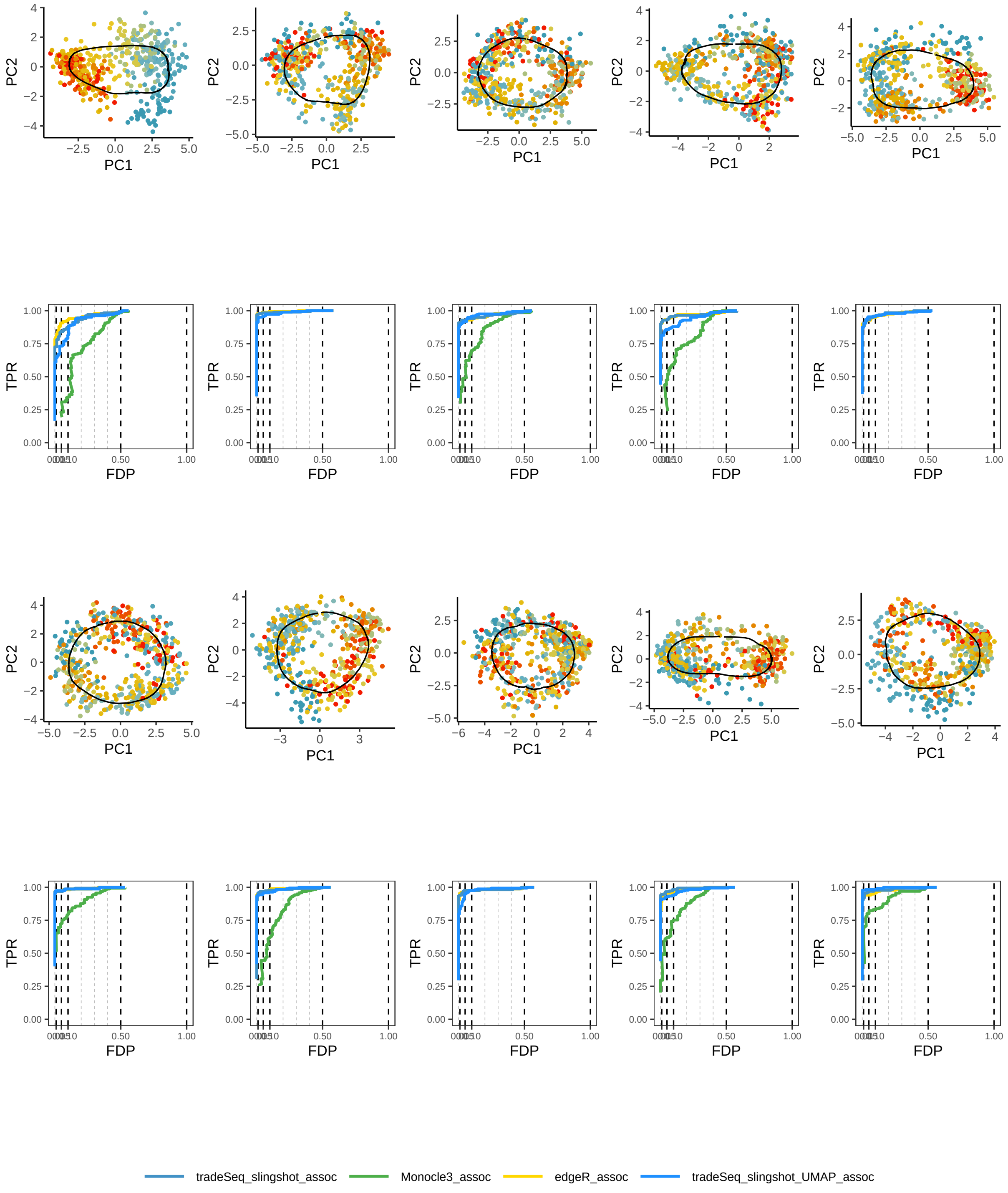

Supplementary Figure 6: *Cyclic simulation scenario: PCA plots with slingshot inferred trajectory and FDP-TPR performance curves for trajectory-based differential expression analysis for each of the 10 simulated datasets. Monocle 3 errored on three datasets. edgeR_assoc is the edgeR-based version of the* `associationTest`.

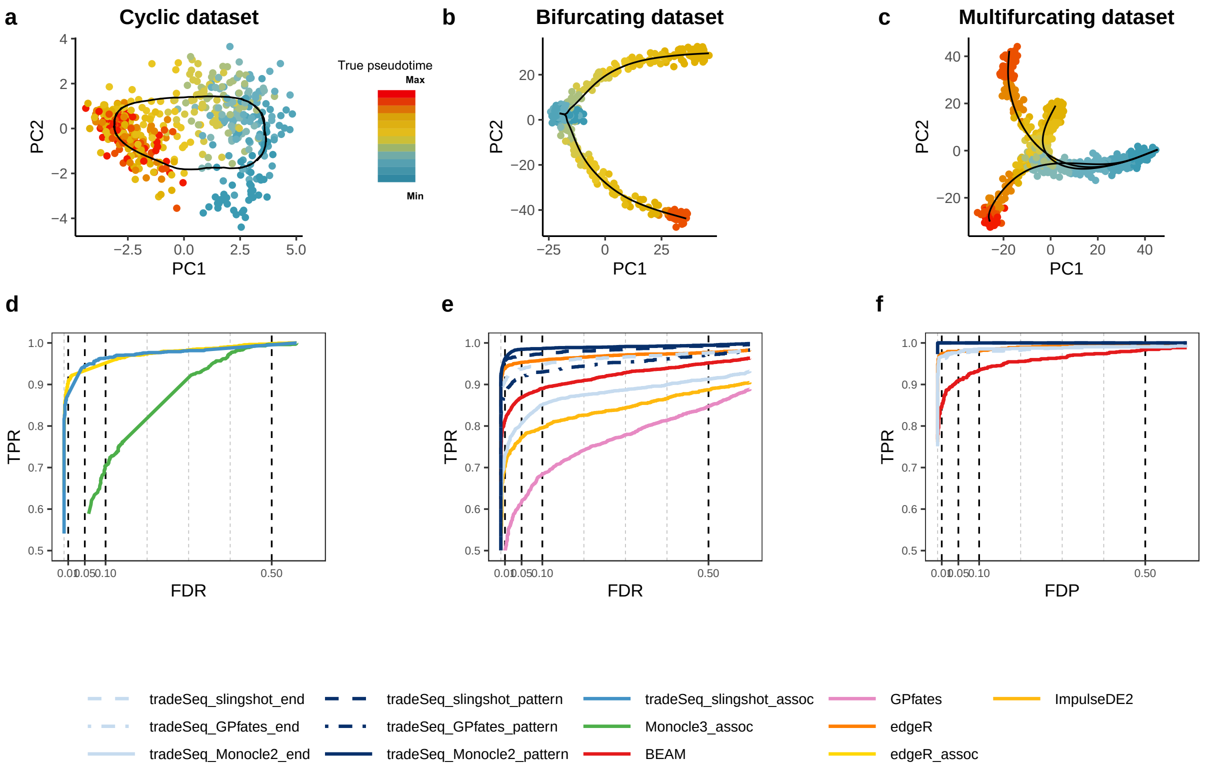

Supplementary Figure 7: *Simulation study results, including* edgeR*-based* `associationTest` *for the cyclic scenario.* PCA plots for the **(a)** cyclic, **(b)** bifurcating, and **(c)** multifurcating simulated trajectories. The plotting symbol for each cell is colored according to its true pseudotime; trajectories (in black) were inferred by princurve in (a) and slingshot in (b) and (c). **(d-f)** Scatterplot of the true positive rate (TPR) vs. the false discovery rate (FDR) or false discovery proportion (FDP) for various DE methods applied to the simulated datasets. Panel (d) displays the average performance curves of DE methods across seven out of 10 cyclic datasets for which all DE methods worked (Monocle 3 errored on three datasets). The `associationTest` from tradeSeq has superior performance for discovering genes whose expression is associated with pseudotime, as compared to Monocle 3. When investigating differential expression between lineages of a trajectory, the `patternTest` of tradeSeq consistently outperforms the `diffEndTest` across all three TI methods, since it is capable of comparing expression across entire lineages. Panel (e) displays the average performance curves across the three bifurcating datasets where all TI methods recovered the correct topology. Here, all tradeSeq `patternTest` workflows, tradeSeq_slingshot_end, and edgeR have similar performance and all are superior to BEAM, ImpulseDE2, and GPfates. Note that the performance of tradeSeq_Monocle2_end deteriorates as compared to tradeSeq_slingshot_end; the curve for tradeSeq_GPfates_end is not visible in this panel due to its low performance. For the multifurcating dataset of panel (f), tradeSeq_slingshot has the highest performance, closely followed by tradeSeq_Monocle2 and edgeR.

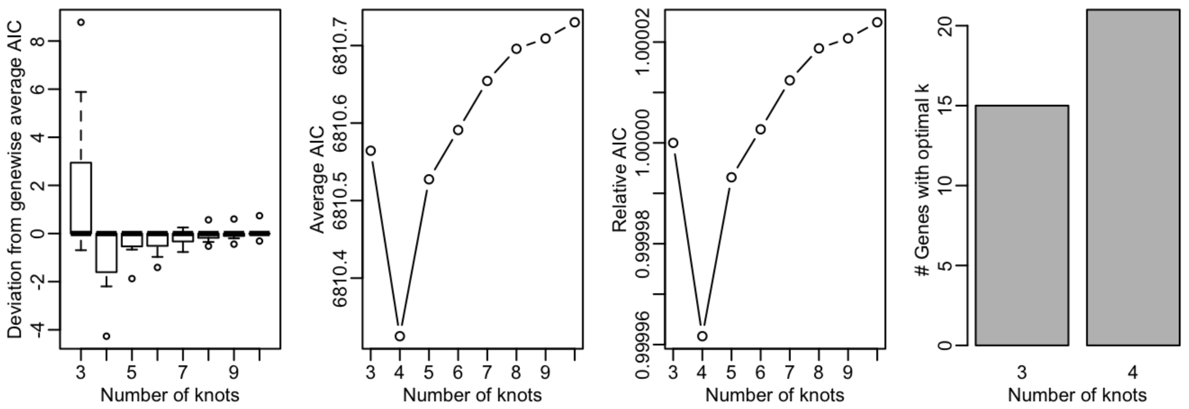

Supplementary Figure 8: *Bifurcating simulation scenario: Selecting the optimal number of knots k using the AIC.* Selecting the optimal number of knots, $k \in \{3, \ldots, 10\}$, using the AIC for a random subset of 250 genes, as implemented in the `evaluateK` function in `tradeSeq`. The left panel shows boxplots (center line, median; box limits, upper and lower quartiles; whiskers, 1.5× interquartile range) of the differences in AIC value with respect to the gene-wise average AIC for the range of k. The middle panels show the evolution of the average AIC (second panel) and relative AIC (third panel) across k. The relative AIC is defined as the relative change with respect to the average AIC at $k = 3$. The barplot in the right panel shows the number of genes which achieve their lowest AIC value for a given k. Here, only genes for which the AIC value varied substantially enough across k (i.e., range in AIC greater than 2) are considered.

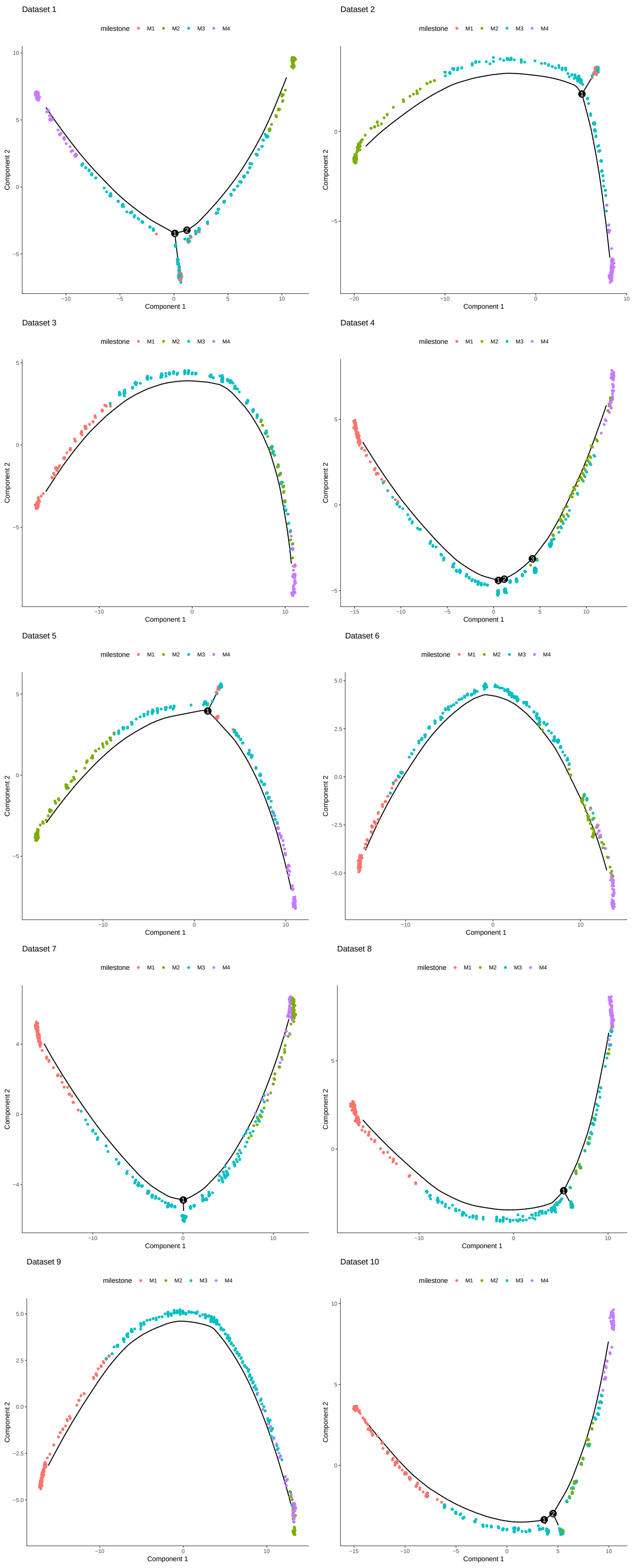

Supplementary Figure 9: *Bifurcating simulation scenario: Monocle 2 inferred trajectories for each of the 10 the simulated datasets.* Cells are plotted in two-dimensional space using DDRTree dimensionality reduction[18]. The simulated trajectory starts at milestone 1 and then continues into milestone 3, generating the two lineages that consist of milestone 2 and milestone 4. The trajectory is correctly recovered in, for example, Dataset 1 (top left panel). Dataset 4, on the other hand, wrongly assigns milestone 2 and milestone 4 to the same lineage, hence failing to recover the true bifurcation point.

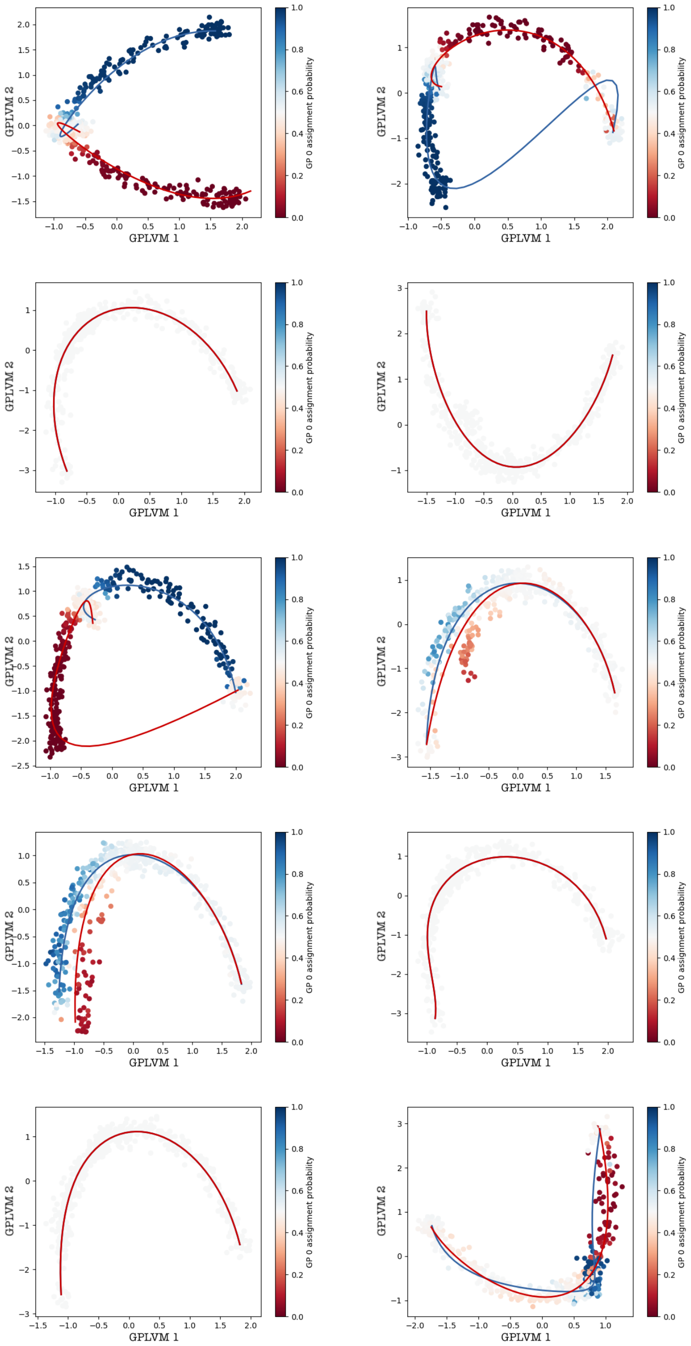

Supplementary Figure 10: *Bifurcating simulation scenario: GPfates inferred trajectories for each of the 10 simulated datasets.* Two-dimensional representation of the datasets for the bifurcating simulation scenario (dynverse toolbox) using Gaussian process latent variable models, as implemented in GPfates. Cells are colored according to their assignment probability to the blue lineage.

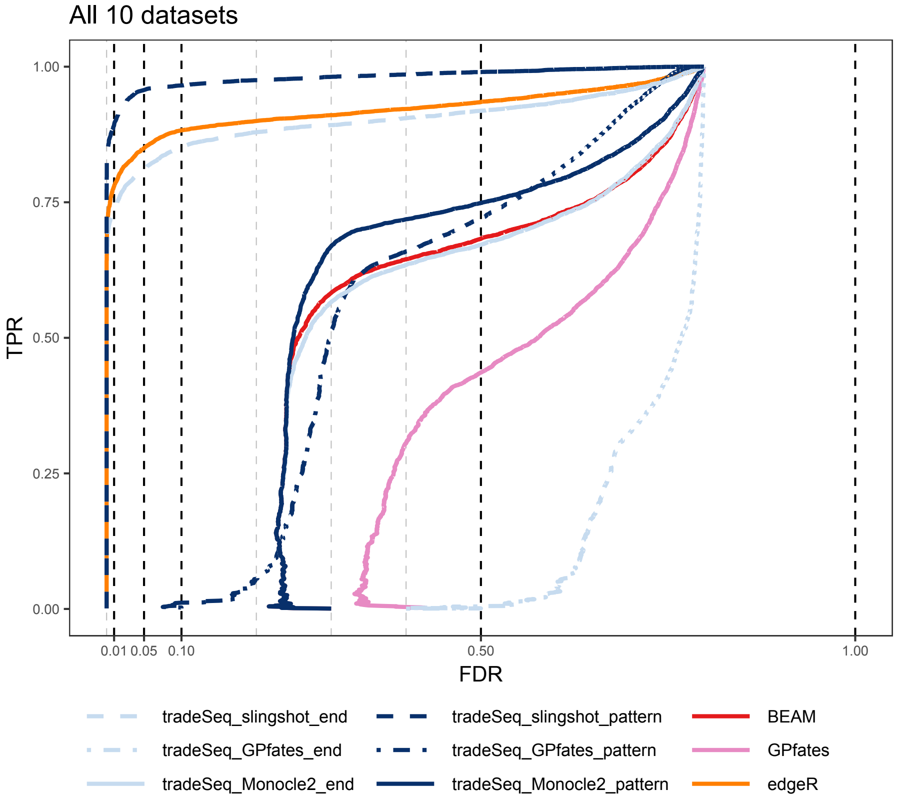

Supplementary Figure 11: *Bifurcating simulation scenario: Mean FDR-TPR performance curves for trajectory-based differential expression analysis across all* 10 *simulated datasets.* ImpulseDE2 is not plotted since we were unable to run it on several datasets.

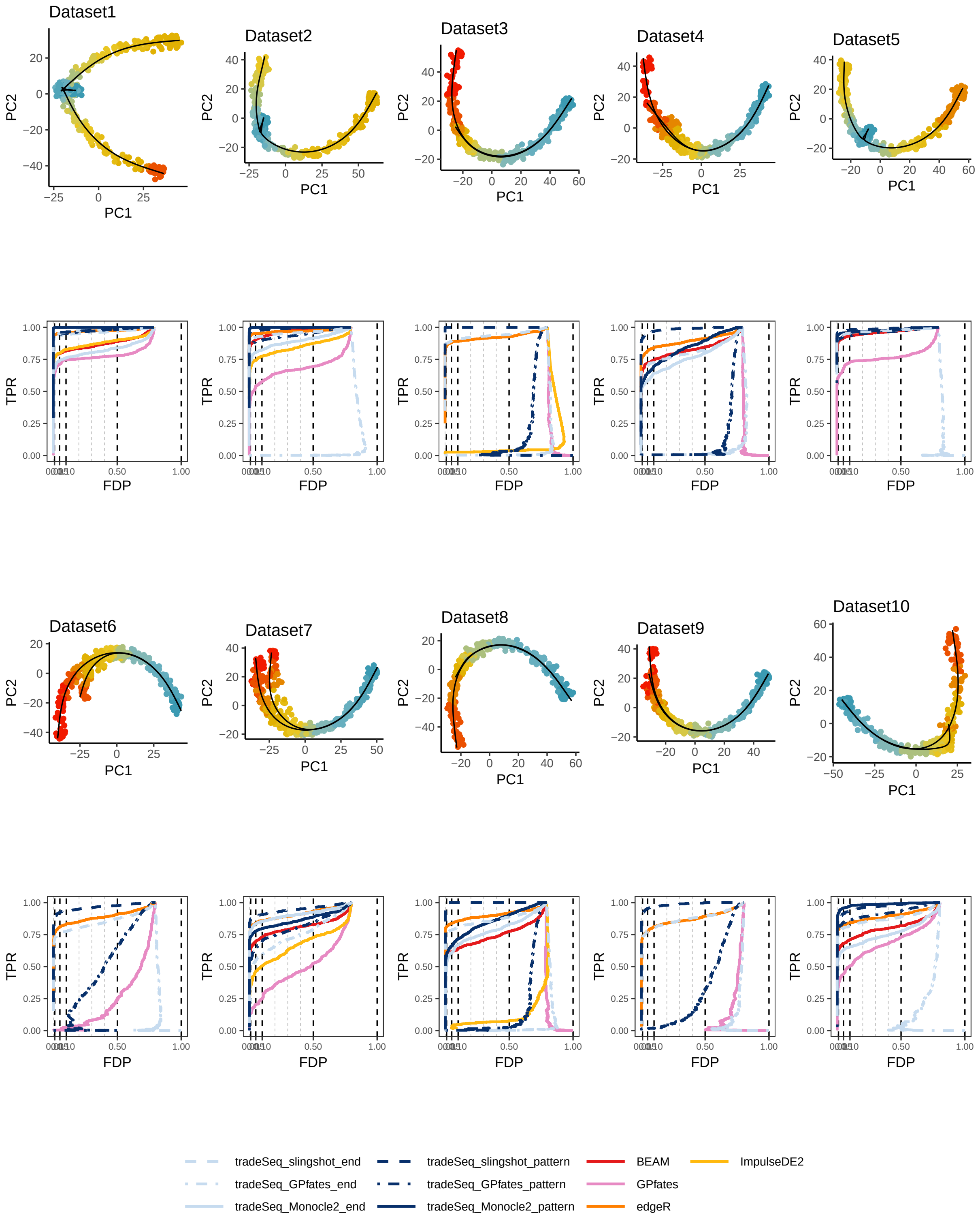

Supplementary Figure 12: *Bifurcating simulation scenario: FDP-TPR performance curves for trajectory-based differential expression analysis for each of the 10 simulated datasets.* Note that the BEAM and tradeSeq_Monocle2 methods are not plotted for Datasets 3, 6, and 9, since Monocle2 failed to discover a branching trajectory for these datasets. We were unable to run ImpulseDE2 on datasets 4, 5, 6, 9, and 10 due to errors.

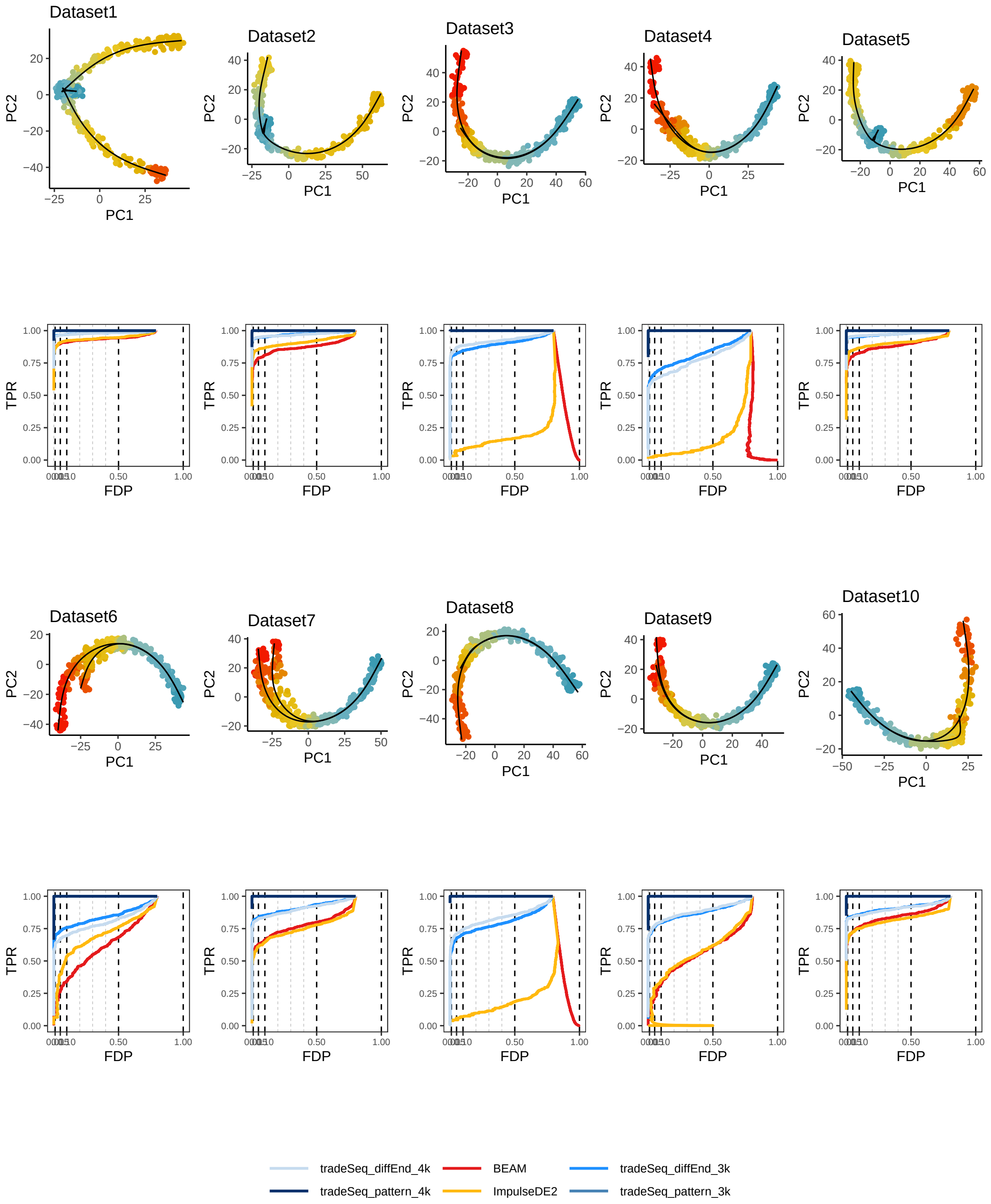

Supplementary Figure 13: *Bifurcating simulation scenario: FDP-TPR performance curves for trajectory-based differential expression analysis based on the simulation ground truth.* To allow a comparison with the BEAM approach, which fits smoothers using 3 knots, we fitted the tradeSeq NB-GAM once with 3 knots and once with 4 knots. We found the latter to provide an optimal fit in terms of AIC. The tradeSeq `patternTest` is unaffected by the number of knots, hence the performance curves overlap. tradeSeq consistently outperforms both BEAM and ImpulseDE2 in all datasets. Note that we did not include the GPfates method in this evaluation, since we were unable to provide the simulation ground truth as input to the method.

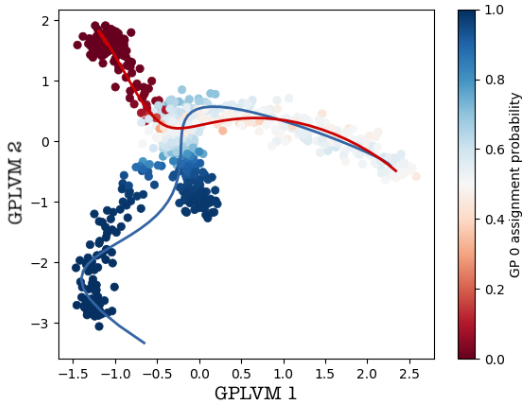

Supplementary Figure 14: *Multifurcating simulation scenario:* `GPfates` *inferred trajectory on one simulated dataset.* Two-dimensional representation of the dataset for the multifurcating simulation scenario using Gaussian process latent variable models, as implemented in `GPfates`. Cells are colored according to their assignment probability to the blue lineage.

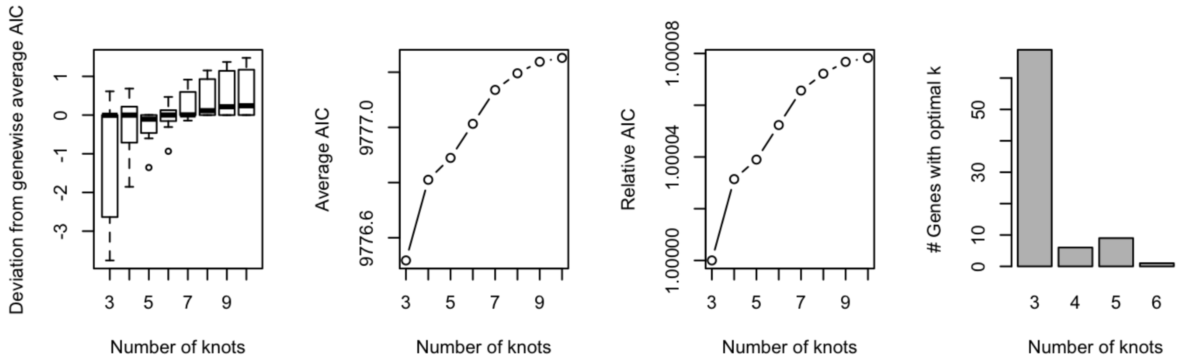

Supplementary Figure 15: *Multifurcating simulation scenario: Selecting the optimal number of knots k using the AIC.* Selecting the optimal number of knots, $k \in \{3, \ldots, 10\}$, using the AIC for a random subset of 500 genes, as implemented in the `evaluateK` function in `tradeSeq`. The left panel shows boxplots (center line, median; box limits, upper and lower quartiles; whiskers, 1.5× interquartile range) of the differences in AIC value with respect to the gene-wise average AIC for the range of k. The middle panels show the evolution of the average AIC (second panel) and relative AIC (third panel) across k. The relative AIC is defined as the relative change with respect to the average AIC at $k = 3$. The barplot in the right panel shows the number of genes which achieve their lowest AIC value for a given k. Here, only genes for which the AIC value varied substantially enough across k (i.e., range in AIC greater than 2) are considered.

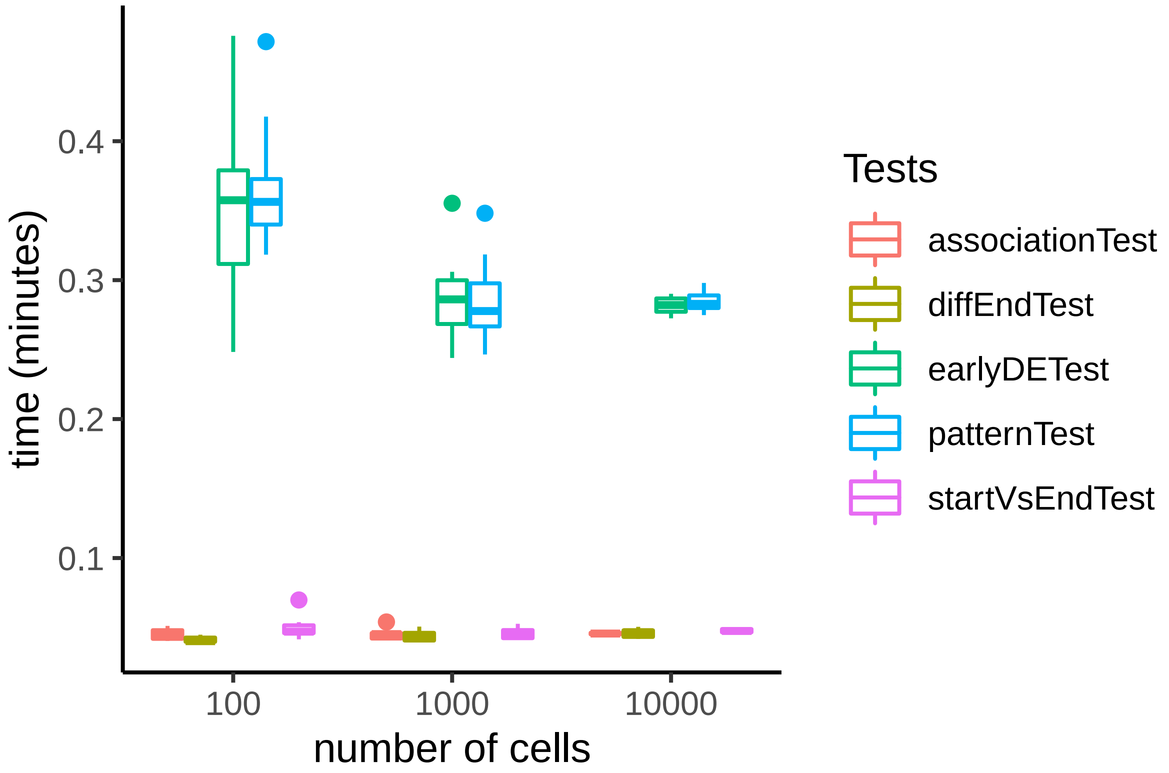

Supplementary Figure 16: *Computational time benchmark for the various tests implemented in* `tradeSeq`*.* Datasets of increasing size (in terms of number of cells) were simulated, each consisting of 5,000 genes. The `fitGAM` function of `tradeSeq` was ran with 4 knots. The computational time required to run the tests for all genes is benchmarked using the `microbenchmark` package, with 10 iterations each. `patternTest` and `earlyDETest` are slower than `associationTest`, `diffEndTest`, and `startVsEndTest`, but all take under 30 seconds to run. The time requirement is constant with respect to the number of cells.

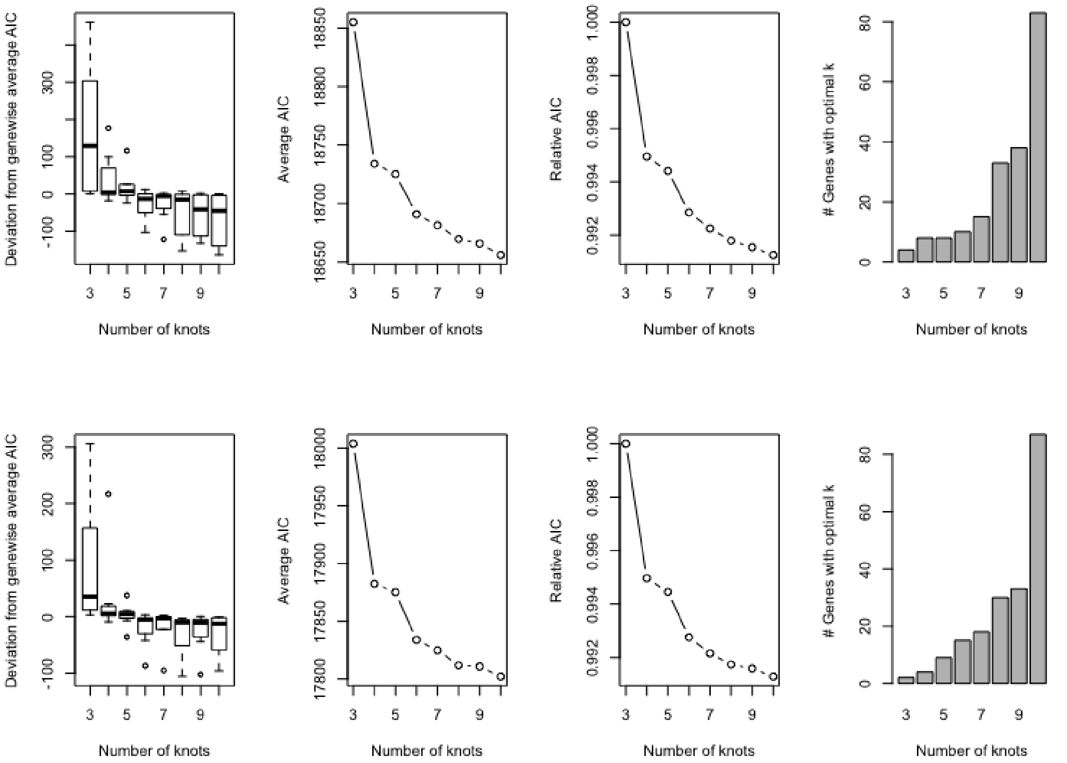

Supplementary Figure 17: *Adipocyte differentiation dataset: Selecting the optimal number of knots k using the AIC.* Selecting the optimal number of knots, $k \in \{3, \ldots, 10\}$, using the AIC for two random subsets (top and bottom rows represent one subset each) of 200 genes, as implemented in the `evaluateK` function in `tradeSeq`. The left panel shows boxplots (center line, median; box limits, upper and lower quartiles; whiskers, 1.5× interquartile range) of the differences in AIC value with respect to the gene-wise average AIC for the range of k. The middle panels show the evolution of the average AIC (second panel) and relative AIC (third panel) across k. The relative AIC is defined as the relative change with respect to the average AIC at $k = 3$. The barplot in the right panel shows the number of genes which achieve their lowest AIC value for a given k. Here, only genes for which the AIC value varied substantially enough across k (i.e., range in AIC greater than 2) are considered.

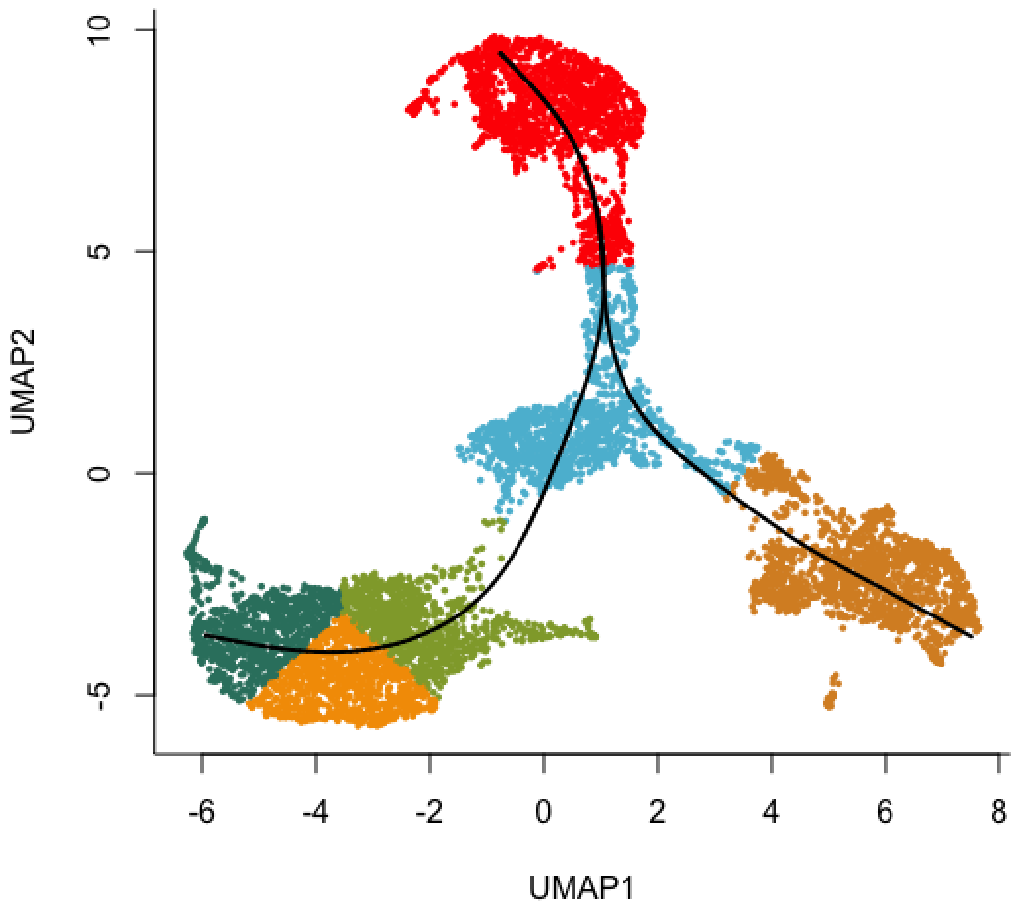

Supplementary Figure 18: *Adipoyte differentiaion dataset: Inferred trajectory.* The scRNA-seq data are plotted in 2D UMAP space, and each cell is colored according to its cluster membership as derived by k-means clustering with $k = 6$ clusters. The black solid line represents the trajectory as estimated by slingshot.

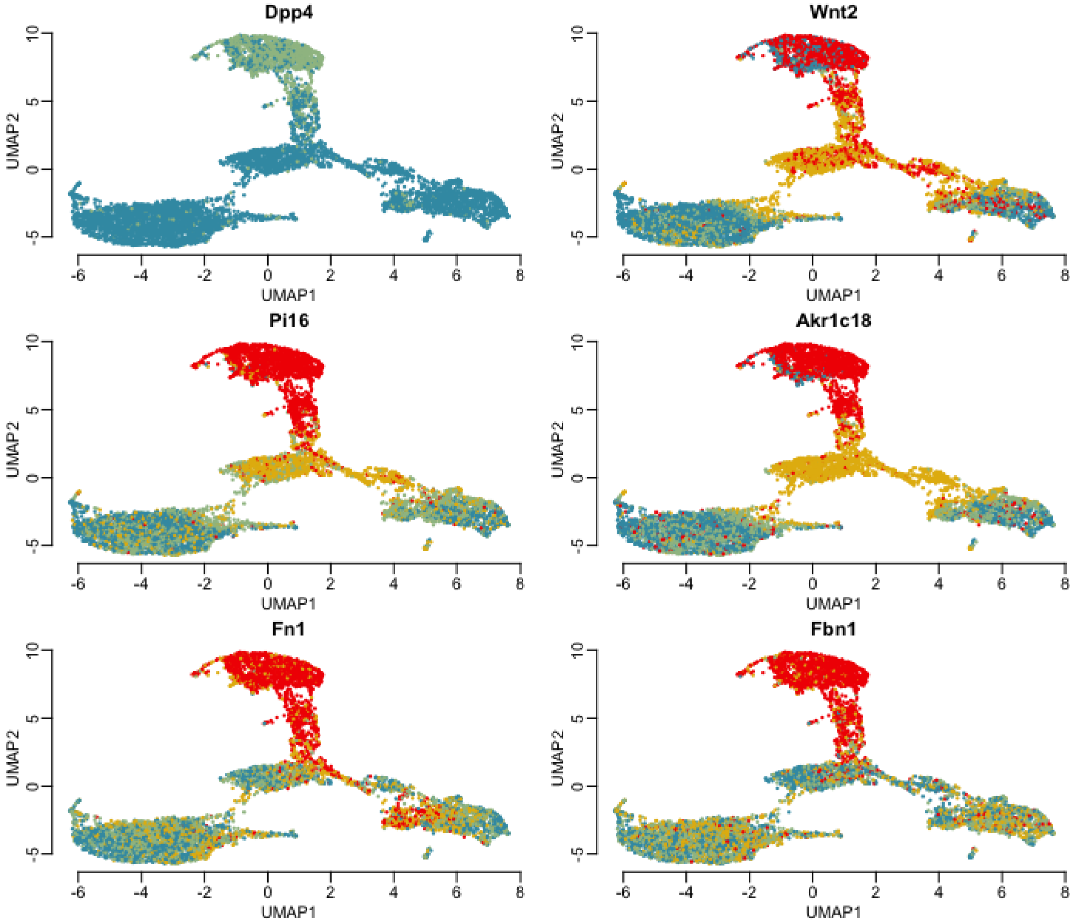

Supplementary Figure 19: *Adipocyte differentiation dataset: Top markers for progenitor cell population.* The scRNA-seq data are plotted in 2D UMAP space, and each cell is colored according to the expression of one of six genes (the expression range is divided into 4 bins, where blue corresponds to low expression and red corresponds to high expression). The top row corresponds to two marker genes, *Dpp4+* and *Wnt2*, from the original manuscript[7]. Other plots are top genes identified with the `startVsEndTest` procedure from `tradeSeq`.

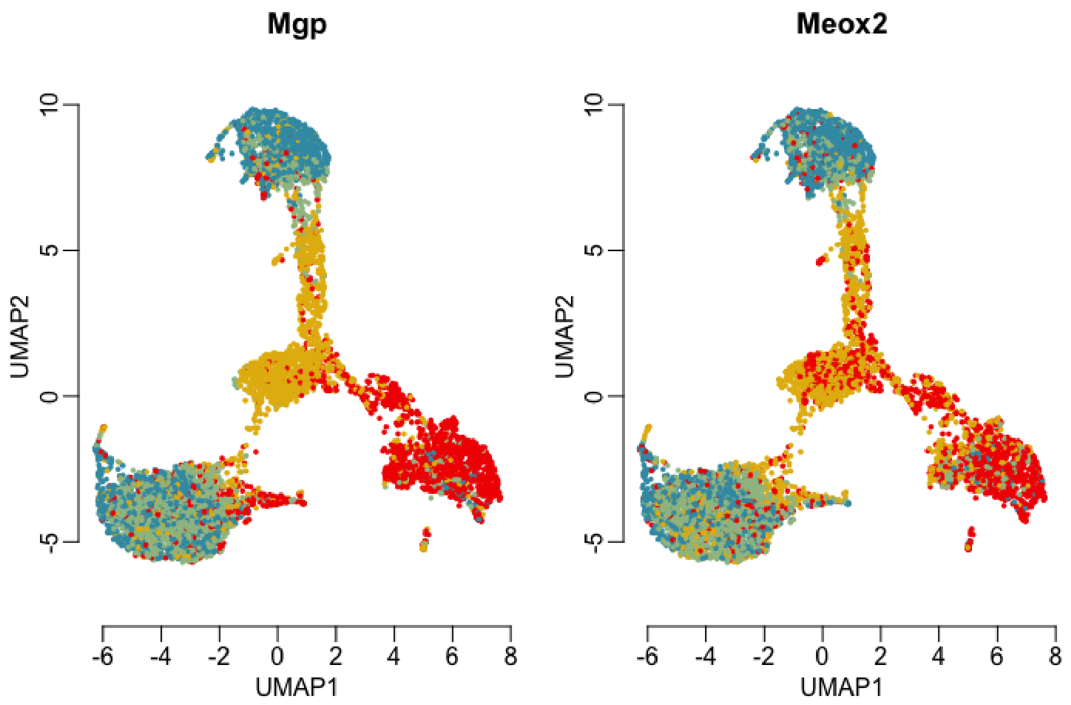

Supplementary Figure 20: *Adipocyte differentiation dataset: Genes upregulated in the adipocyte precursor stage and a single differentiated cell type.* The scRNA-seq data are plotted in 2D UMAP space, and each cell is colored according to the expression of one of two genes (the expression range is divided into 4 bins, where blue corresponds to low expression and red corresponds to high expression).

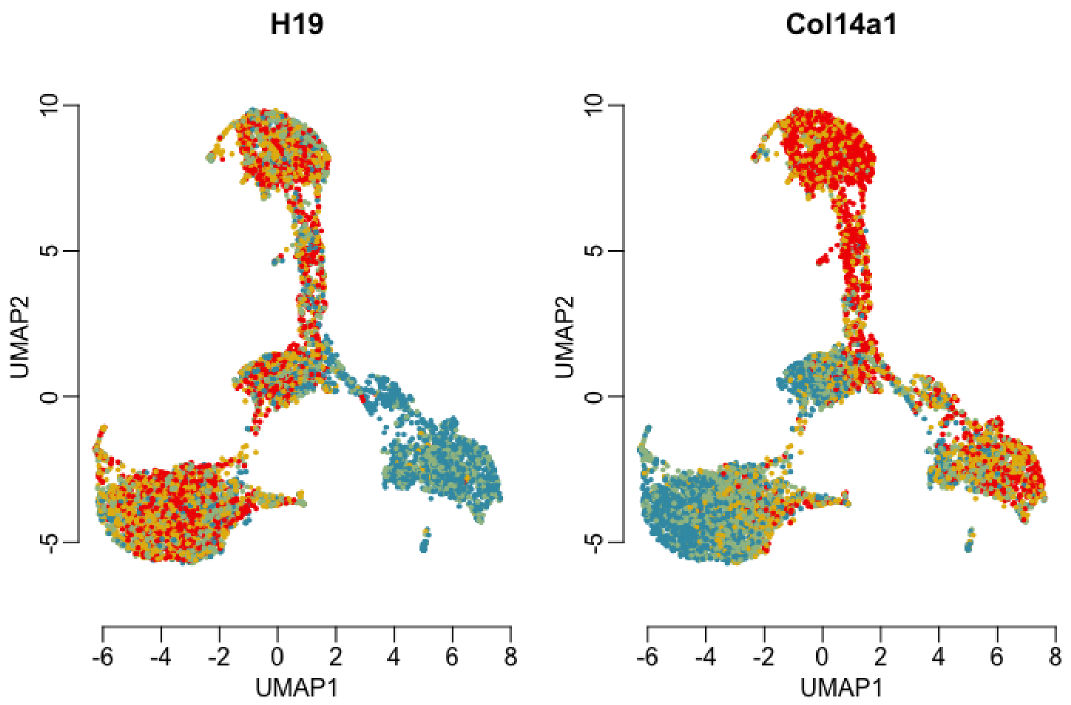

Supplementary Figure 21: *Adipocyte differentiation dataset: Genes sporadically upregulated across the entire lineage for a single differentiated cell type.* The scRNA-seq data are plotted in 2D UMAP space, and each cell is colored according to the expression of one of two genes (the expression range is divided into 4 bins, where blue corresponds to low expression and red corresponds to high expression).

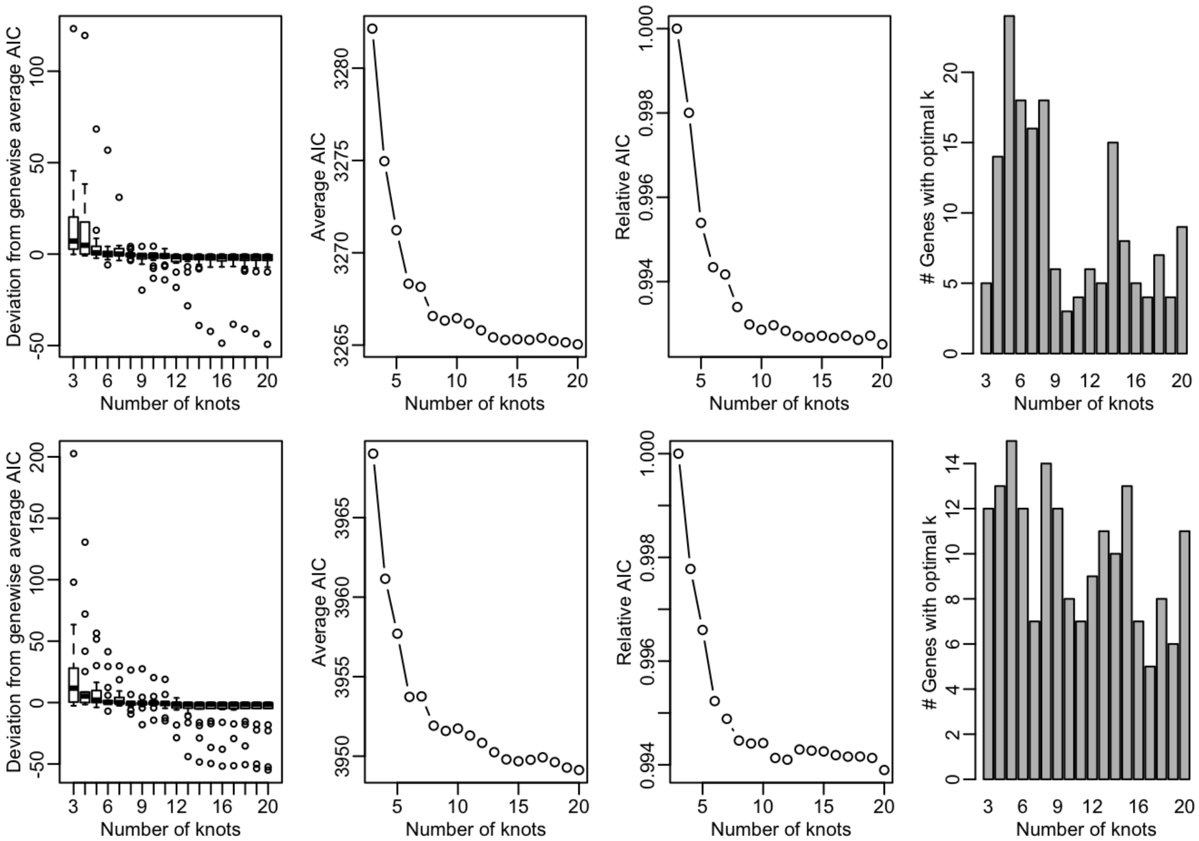

Supplementary Figure 22: *Mouse bone marrow dataset: Selecting the optimal number of knots k using the AIC.* Selecting the optimal number of knots, $k \in \{3, \ldots, 20\}$, using the AIC for two random subsets (top and bottom rows represent one subset each) of 250 genes, as implemented in the `evaluateK` function in `tradeSeq`. The left panel shows boxplots (center line, median; box limits, upper and lower quartiles; whiskers, 1.5× interquartile range) of the differences in AIC value with respect to the gene-wise average AIC for the range of k. The middle panels show the evolution of the average AIC (second panel) and relative AIC (third panel) across k. The relative AIC is defined as the relative change with respect to the average AIC at $k = 3$. The barplot in the right panel shows the number of genes which achieve their lowest AIC value for a given k. Here, only genes for which the AIC value varied substantially enough across k (i.e., range in AIC greater than 2) are considered.

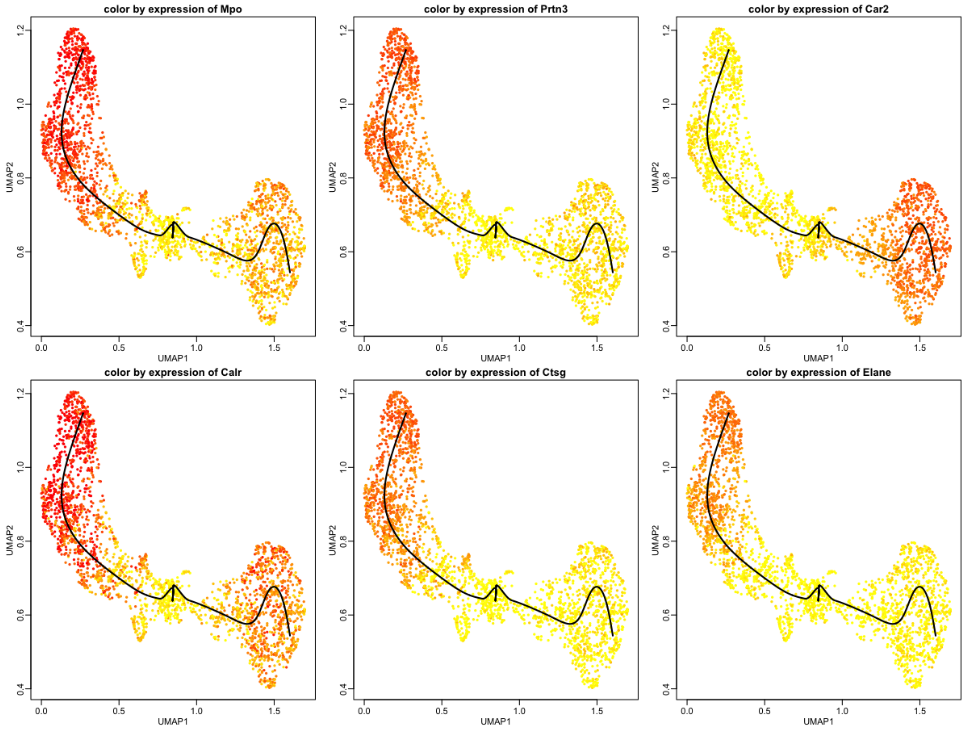

Supplementary Figure 23: *Mouse bone marrow dataset:* ***tradeSeq*** *recovers markers for the progenitor cell population.* This figure shows the six most significant genes when testing for differential expression between the progenitor cell type (i.e., starting point of the smoother) and differentiated cell types (i.e., endpoint of the smoother) for the data from Paul et al.[4] using the `startVsEndTest` in `tradeSeq`. Yellow denotes low expression, while red denotes high expression.

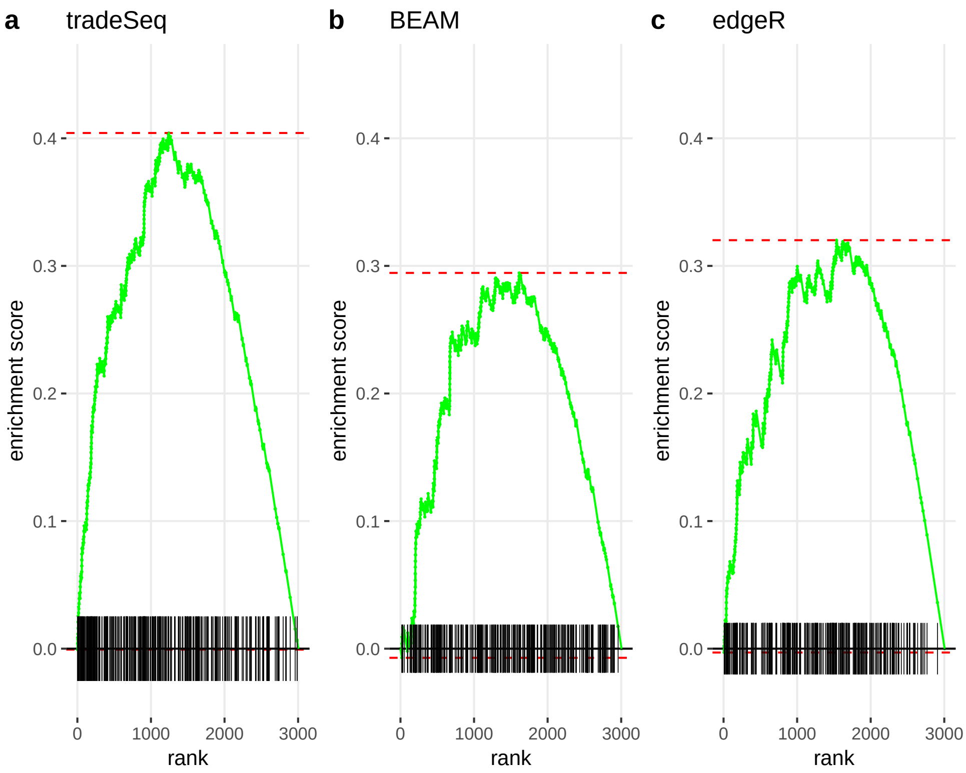

Supplementary Figure 24: *Mouse bone marrow dataset: Gene set enrichment plots for the erythrocyte gene set from de Graaf et al.*[8]*, for three differential expression methods, tradeSeq, BEAM, and edgeR.* Enrichment is determined for each method based on its respective ranking of the genes according to evidence for differential expression. Genes that are contained in the erythrocyte gene set are denoted with vertical lines at the bottom of each figure, and the green curve represents the gene enrichment score along the gene rankings. tradeSeq has the highest enrichment score, as determined by the dashed red line, since genes that are related to erythrocytes predominantly have high rankings for differential expression, while the distribution of erythrocyte genes seems more uniform with, for example, the BEAM approach.

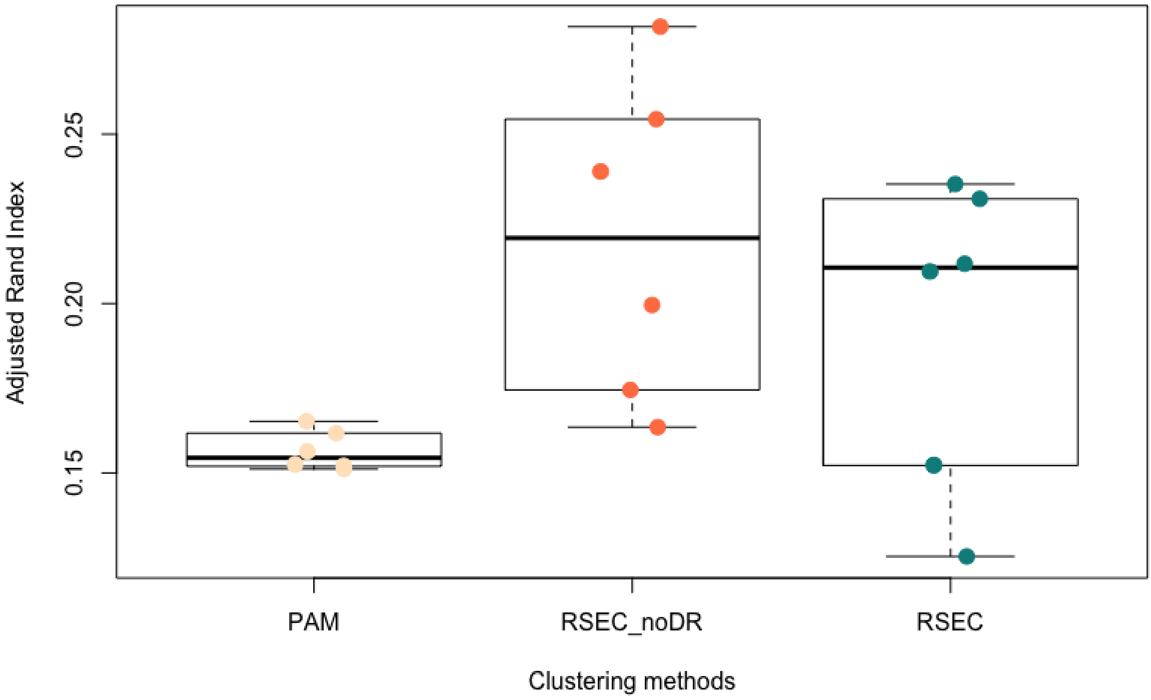

Supplementary Figure 25: *Mouse bone marrow dataset: Stability of gene clustering methods across bootstrap samples.* Boxplots (center line, median; box limits, upper and lower quartiles; whiskers, 1.5× interquartile range) of gene clustering stability measures are shown for three clustering algorithms. The stability of a gene clustering method is evaluated using non-parametric bootstrapping of the cells (for computational reasons, we restricted this evaluation to six bootstrap samples). We consider all genes found to be significant at a 5% nominal FDR level by `patternTest` for the dataset of Paul et al.[4]. For each bootstrap sample, cells are sampled at random with replacement, the NB-GAM is refit using tradeSeq, and genes are clustered based on the tradeSeq fitted values, using both partitioning around medoids (PAM) and RSEC. We compare RSEC against PAM since the latter is also used in Monocle for gene clustering. For RSEC, we evaluate both clustering on the fitted values directly (method 'RSEC_noDR' in the figure) as well as clustering after dimensionality reduction with principal component analysis (the default for RSEC, as implemented in clusterExperiment, with automatic determination of the number of principal components; method 'RSEC' in the figure). The stability of the clustering is evaluated by comparing the bootstrapped clusterings with the original clustering based on the full dataset using the adjusted Rand index (ARI)[19].

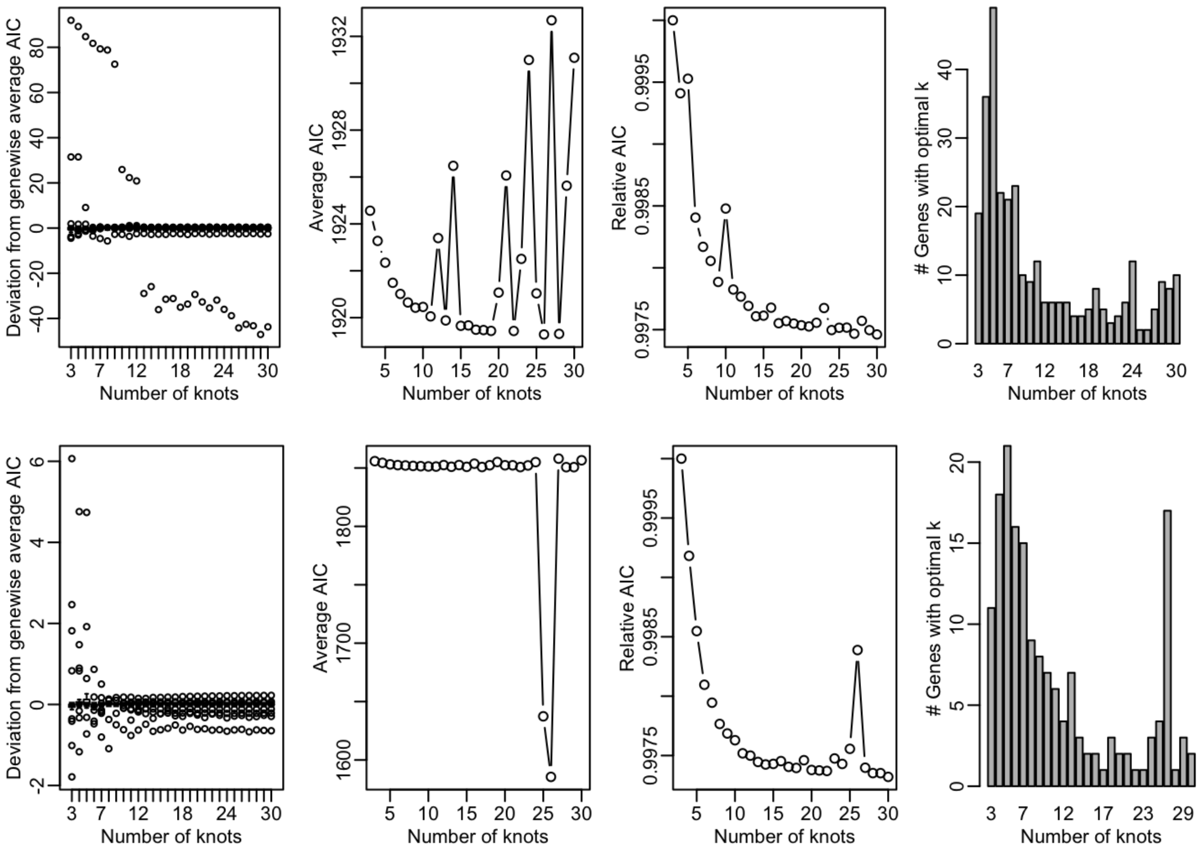

Supplementary Figure 26: *Olfactory epithelium dataset: Selecting the optimal number of knots k using the AIC.* Selecting the optimal number of knots, $k \in \{3, \ldots, 30\}$, using the AIC for two random subsets (top and bottom rows represent one subset each) of $1,000$ genes, as implemented in the `evaluateK` function in `tradeSeq`. The left panel shows boxplots (center line, median; box limits, upper and lower quartiles; whiskers, $1.5\times$ interquartile range) of the differences in AIC value with respect to the gene-wise average AIC for the range of k. The middle panels show the evolution of the average AIC (second panel) and relative AIC (third panel) across k. The relative AIC is defined as the relative change with respect to the average AIC at $k = 3$. The barplot in the right panel shows the number of genes which achieve their lowest AIC value for a given k. Here, only genes for which the AIC value varied substantially enough across k (i.e., range in AIC greater than 2) are considered.

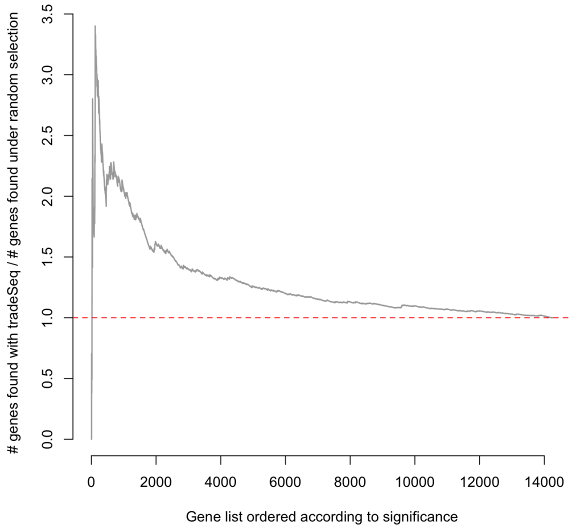

Supplementary Figure 27: *Mouse olfactory epithelium dataset: Cell cycle genes in the neuronal lineage.* The figure illustrates the enrichment of cell cyle genes in lists of genes whose expression was found to be most significantly associated with the neuronal lineage according to the `associationTest` procedure in `tradeSeq`. The list of cell cycle related genes was obtained from the Mouse Genome Informatics (MGI) website at `http://www.informatics.jax.org/go/term/GO:0007049`. On the x-axis, genes are ordered according to their significance based on `associationTest`. The y-axis shows the ratio of the number of cell cycle genes among a set of top significant genes relative to the number of cell cycle genes one would expect by chance (i.e., if DE genes were randomly found/sampled). Specifically, if we let C denote the proportion of genes associated with the cell cycle according to the MGI database, then, under the hypothesis that cell cycle genes are randomly discovered as DE, the expected number of cell cycle genes in the list of top N genes is NC. The relative number that is plotted on the y-axis is then the ratio between the number of cell cycle genes discovered by `tradeSeq` for a given top list of size N and NC.

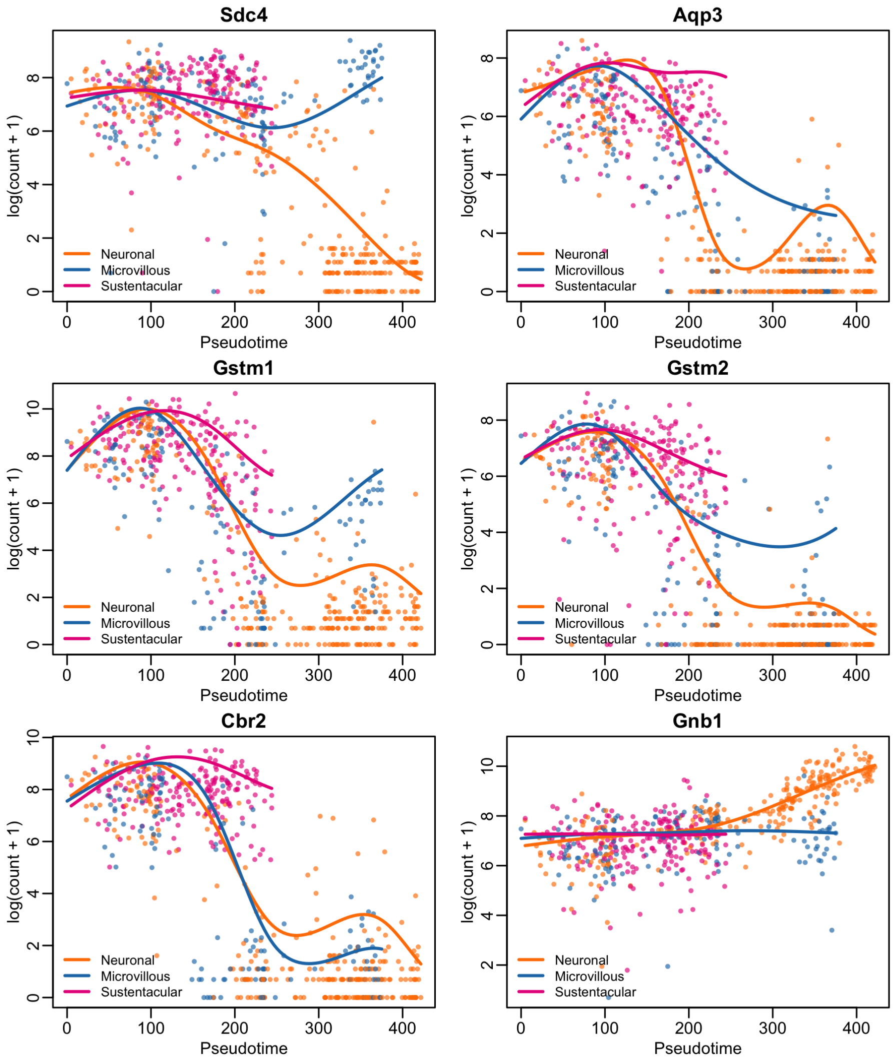

Supplementary Figure 28: *Mouse olfactory epithelium dataset: Top six differentially expressed genes as identified by a ZINB analysis with* `tradeSeq patternTest`*.* Every lineage is represented by a smooth function of gene expression along pseudotime. The cells assigned to a particular lineage based on the slingshot weights are represented with the same color as the lineage.

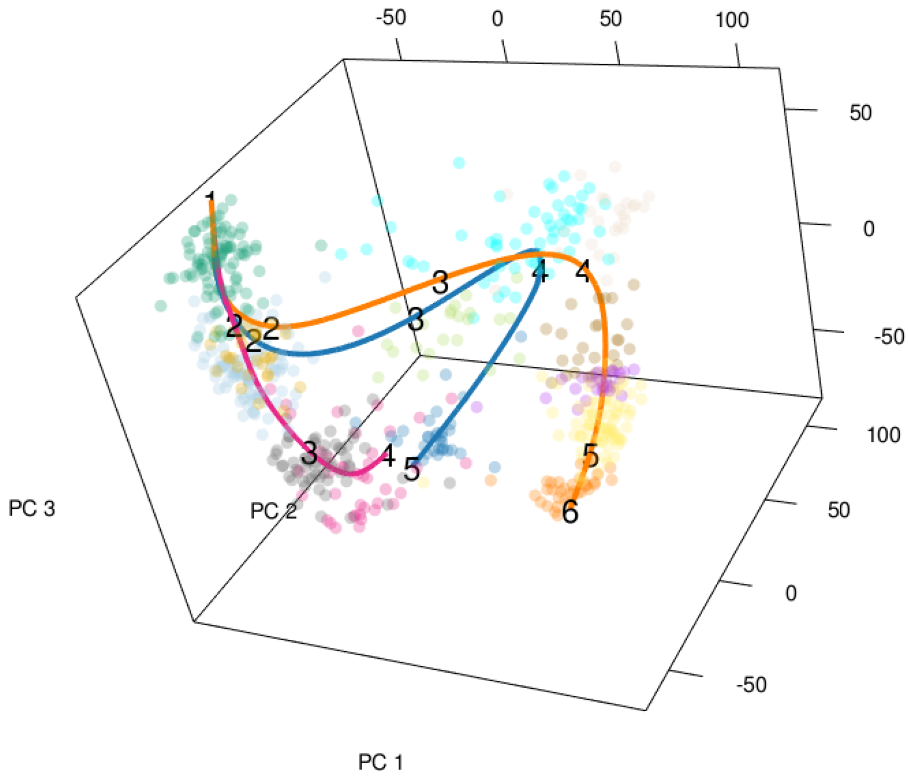

Supplementary Figure 29: *Mouse olfactory epithelium dataset: Trajectory with knots.* Three-dimensional PCA plot of the scRNA-seq data from Fletcher et al. [20], where cells are colored according to their cluster membership as defined in the original paper (see Methods). The simultaneous principal curves for the lineages inferred by slingshot are displayed. The numbers on each lineage specify the knot points used to fit the ZINB-GAM in tradeSeq. The first branching event occurs between knots 1 and 3.

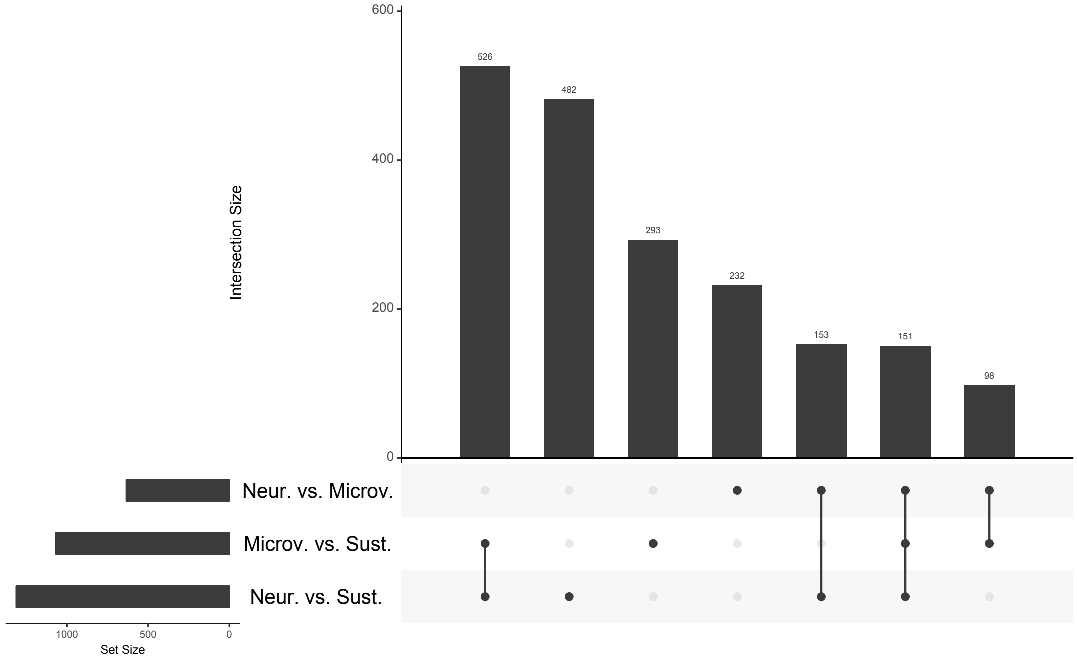

Supplementary Figure 30: *Mouse olfactory epithelium dataset: UpSet plot for `earlyDETest` applied around the first branching point to each pair among the three lineages.* Left panel: Barplot of the number of DE genes for each contrast/pair (5% nominal FDR level). Top panel: Barplot of the number of DE genes for one, two of the three, or all three contrasts. Center panel: Contrasts being considered for the top panel. For example, the first column shows that 526 genes are DE for both the Microv vs. Sust contrast and the Neur. vs. Sust contrast.

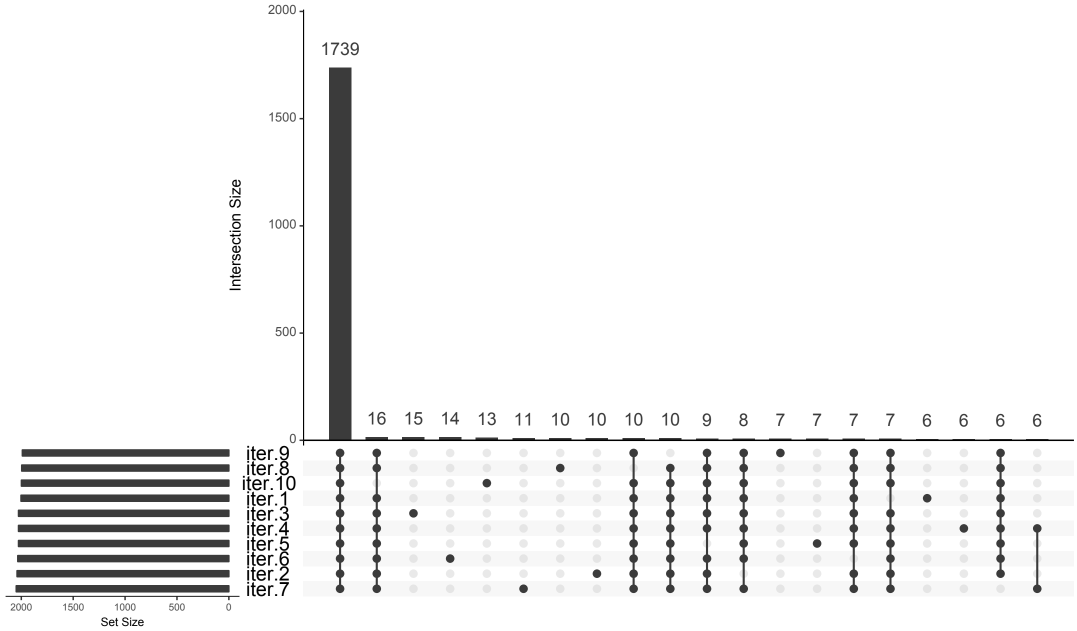

Supplementary Figure 31: *Mouse bone marrow dataset: UpSet plot for `startVsEndTest`, for multiple assignments of cells to lineages.* Ten random multinomial assignments of cells to lineages are performed and denoted with 'iter.1' to 'iter.10'. Left panel: Barplot of the number of DE genes for each assignment (5% nominal FDR level). Top panel: Barplot of the number of DE genes in common for various combinations of the 10 assignments. Center panel: Assignments being considered for the top panel. Only the 20 combinations with the largest number of common genes are shown in the figure.

References

[1] GK Smyth. Linear models and empirical bayes methods for assessing differential expression in microarray experiments. *Statistical applications in genetics and molecular biology*, 3(1):1–26, 2004. URL http://www.degruyter.com/view/j/sagmb.2004.3.1/sagmb.2004.3.1.1027/sagmb.2004.3.1.1027.xml.

[2] Kelly Street, Davide Risso, Russell B. Fletcher, Diya Das, John Ngai, Nir Yosef, Elizabeth Purdom, and Sandrine Dudoit. Slingshot: cell lineage and pseudotime inference for single-cell transcriptomics. *BMC Genomics*, 19(1):477, dec 2018. ISSN 1471-2164. doi: 10.1186/s12864-018-4772-0. URL https://bmcgenomics.biomedcentral.com/articles/10.1186/s12864-018-4772-0.

[3] Tapio Lönnberg, Valentine Svensson, Kylie R James, Daniel Fernandez-Ruiz, Ismail Sebina, Ruddy Montandon, Megan S F Soon, Lily G Fogg, Arya Sheela Nair, Urijah Liligeto, Michael J T Stubbington, Lam-Ha Ly, Frederik Otzen Bagger, Max Zwiessele, Neil D Lawrence, Fernando Souza-Fonseca-Guimaraes, Patrick T Bunn, Christian R Engwerda, William R Heath, Oliver Billker, Oliver Stegle, Ashraful Haque, and Sarah A Teichmann. Single-cell RNA-seq and computational analysis using temporal mixture modelling resolves Th1/Tfh fate bifurcation in malaria. *Science immunology*, 2(9), mar 2017. doi: 10.1126/sciimmunol.aal2192. URL http://www.ncbi.nlm.nih.gov/pubmed/28345074.

[4] Franziska Paul, Ya'ara Arkin, Amir Giladi, Diego Adhemar Jaitin, Ephraim Kenigsberg, Hadas Keren-Shaul, Deborah Winter, David Lara-Astiaso, Meital Gury, Assaf Weiner, Eyal David, Nadav Cohen, Felicia Kathrine Bratt Lauridsen, Simon Haas, Andreas Schlitzer, Alexander Mildner, Florent Ginhoux, Steffen Jung, Andreas Trumpp, Bo Torben Porse, Amos Tanay, and Ido Amit. Transcriptional Heterogeneity and Lineage Commitment in Myeloid Progenitors. *Cell*, 163(7):1663–1677, dec 2015. ISSN 0092-8674. doi: 10.1016/J.CELL.2015.11.013. URL https://www.sciencedirect.com/science/article/pii/S0092867415014932?via{%}3Dihub{#}app3.

[5] Vladimir Yu. Kiselev, Veronique Juvin, Mouhannad Malek, Nicholas Luscombe, Phillip Hawkins, Nicolas Le Novère, and Len Stephens. Perturbations of PIP3 signalling trigger a global remodelling of mRNA landscape and reveal a transcriptional feedback loop. *Nucleic Acids Research*, 43(20): gkv1015, oct 2015. ISSN 0305-1048. doi: 10.1093/nar/gkv1015. URL https://academic.oup.com/nar/article-lookup/doi/10.1093/nar/gkv1015.

[6] Yoav Benjamini and Yosef Hochberg. Controlling the False Discovery Rate: A Practical and Powerful Approach to Multiple Testing. *Journal of the Royal Statistical Society. Series B (Methodological)*, 57(1):289–300, 1995.

[7] David Merrick, Alexander Sakers, Zhazira Irgebay, Chihiro Okada, Catherine Calvert, Michael P Morley, Ivona Percec, and Patrick Seale. Identification of a mesenchymal progenitor cell hierarchy in adipose tissue. *Science (New York, N.Y.)*, 364(6438), apr 2019. ISSN 1095-9203. doi: 10.1126/science.aav2501. URL http://www.ncbi.nlm.nih.gov/pubmed/31023895.

[8] Carolyn A. de Graaf, Jarny Choi, Tracey M. Baldwin, Jessica E. Bolden, Kirsten A. Fairfax, Aaron J. Robinson, Christine Biben, Clare Morgan, Kerry Ramsay, Ashley P. Ng, Maria Kauppi, Elizabeth A. Kruse, Tobias J. Sargeant, Nick Seidenman, Angela D'Amico, Marthe C. D'Ombrain, Erin C. Lucas, Sandra Koernig, Adriana Baz Morelli, Michael J. Wilson, Steven K. Dower, Brenda Williams, Shen Y. Heazlewood, Yifang Hu, Susan K. Nilsson, Li Wu, Gordon K. Smyth, Warren S. Alexander, and Douglas J. Hilton. Haemopedia: An Expression Atlas of Murine Hematopoietic Cells. *Stem Cell Reports*, 7(3):571–582, sep 2016. ISSN 22136711. doi: 10.1016/j.stemcr.2016.07.007. URL http://www.ncbi.nlm.nih.gov/pubmed/27499199.

[9] Alexey Sergushichev. An algorithm for fast preranked gene set enrichment analysis using cumulative statistic calculation. *Preprint at https://www.biorxiv.org/content/10.1101/060012v1*, page 060012, jun 2016. doi: 10.1101/060012.

[10] Davis J McCarthy, Yunshun Chen, and Gordon K Smyth. Differential expression analysis of multifactor RNA-Seq experiments with respect to biological variation. *Nucleic acids research*, 40(10): 4288–97, may 2012. ISSN 1362-4962. doi: 10.1093/nar/gks042. URL http://www.pubmedcentral.nih.gov/articlerender.fcgi?artid=3378882{&}tool=pmcentrez{&}rendertype=abstract.

[11] Daisuke Kurotaki, Michio Yamamoto, Akira Nishiyama, Kazuhiro Uno, Tatsuma Ban, Motohide Ichino, Haruka Sasaki, Satoko Matsunaga, Masahiro Yoshinari, Akihide Ryo, Masatoshi Nakazawa, Keiko Ozato, and Tomohiko Tamura. IRF8 inhibits C/EBPα activity to restrain mononuclear phagocyte progenitors from differentiating into neutrophils. *Nature Communications*, 5(1):4978, dec 2014. ISSN 2041-1723. doi: 10.1038/ncomms5978. URL http://www.nature.com/articles/ncomms5978.

[12] Dong-Mi Shin, Chang-Hoon Lee, and Herbert C. Morse. IRF8 Governs Expression of Genes Involved in Innate and Adaptive Immunity in Human and Mouse Germinal Center B Cells. *PLoS ONE*, 6 (11):e27384, nov 2011. ISSN 1932-6203. doi: 10.1371/journal.pone.0027384. URL http://dx.plos.org/10.1371/journal.pone.0027384.

[13] Jean-François Marquis, Oxana Kapoustina, David Langlais, Rebecca Ruddy, Catherine Rosa Dufour, Bae-Hoon Kim, John D. MacMicking, Vincent Giguère, and Philippe Gros. Interferon Regulatory Factor 8 Regulates Pathways for Antigen Presentation in Myeloid Cells and during Tuberculosis. *PLoS Genetics*, 7(6):e1002097, jun 2011. ISSN 1553-7404. doi: 10.1371/journal.pgen.1002097. URL http://dx.plos.org/10.1371/journal.pgen.1002097.

[14] Andrew J. Murphy, Mani Akhtari, Sonia Tolani, Tamara Pagler, Nora Bijl, Chao-Ling Kuo, Mi Wang, Marie Sanson, Sandra Abramowicz, Carrie Welch, Andrea E. Bochem, Jan Albert Kuivenhoven, Laurent Yvan-Charvet, and Alan R. Tall. ApoE regulates hematopoietic stem cell proliferation, monocytosis, and monocyte accumulation in atherosclerotic lesions in mice. *Journal of Clinical Investigation*, 121(10):4138–4149, oct 2011. ISSN 0021-9738. doi: 10.1172/JCI57559. URL http://www.jci.org/articles/view/57559.

[15] Koen Van den Berge, Fanny Perraudeau, Charlotte Soneson, Michael I. Love, Davide Risso, Jean-Philippe Vert, Mark D. Robinson, Sandrine Dudoit, and Lieven Clement. Observation weights unlock bulk RNA-seq tools for zero inflation and single-cell applications. *Genome Biology*, 19(1): 24, dec 2018. ISSN 1474-760X. doi: 10.1186/s13059-018-1406-4. URL https://genomebiology.biomedcentral.com/articles/10.1186/s13059-018-1406-4.

[16] Koen Van den Berge, Charlotte Soneson, Mark D. Robinson, and Lieven Clement. stageR: a general stage-wise method for controlling the gene-level false discovery rate in differential expression and differential transcript usage. *Genome Biology*, 18(1):151, 2017. ISSN 1474-760X. doi: 10.1186/s13059-017-1277-0. URL http://www.ncbi.nlm.nih.gov/pubmed/28784146.

[17] Konika Chawla, Sushil Tripathi, Liv Thommesen, Astrid Lægreid, and Martin Kuiper. TFcheckpoint: a curated compendium of specific DNA-binding RNA polymerase II transcription factors. *Bioinformatics*, 29(19):2519–2520, oct 2013. ISSN 1460-2059. doi: 10.1093/bioinformatics/btt432. URL https://academic.oup.com/bioinformatics/article-lookup/doi/10.1093/bioinformatics/btt432.

[18] Xiaojie Qiu, Qi Mao, Ying Tang, Li Wang, Raghav Chawla, Hannah A Pliner, and Cole Trapnell. Reversed graph embedding resolves complex single-cell trajectories. *Nature Methods*, aug 2017. doi: 10.1038/nmeth.4402. URL https://www.nature.com/nmeth/journal/vaop/ncurrent/full/nmeth.4402.html.

[19] Lawrence Hubert and Phipps Arabie. Comparing partitions. *Journal of Classification*, 2(1):193–218, dec 1985. ISSN 0176-4268. doi: 10.1007/BF01908075. URL http://link.springer.com/10.1007/BF01908075.

[20] Russell B. Fletcher, Diya Das, Levi Gadye, Kelly N. Street, Ariane Baudhuin, Allon Wagner, Michael B. Cole, Quetzal Flores, Yoon Gi Choi, Nir Yosef, Elizabeth Purdom, Sandrine Dudoit, Davide Risso, and John Ngai. Deconstructing Olfactory Stem Cell Trajectories at Single-Cell Resolution. *Cell Stem Cell*, 20(6):817–830.e8, jun 2017. ISSN 19345909. doi: 10.1016/j.stem.2017.04.003. URL http://www.ncbi.nlm.nih.gov/pubmed/28506465.
